# Path planning of mobile robot based on improved double deep Q-network algorithm

**DOI:** 10.3389/fnbot.2025.1512953

**Published:** 2025-02-13

**Authors:** Zhenggang Wang, Shuhong Song, Shenghui Cheng

**Affiliations:** College of Electrical Engineering, Anhui Polytechnic University, Wuhu, China

**Keywords:** deep reinforcement learning, mobile robot, path planning, BiLSTM, Dueling Network

## Abstract

Aiming at the problems of slow network convergence, poor reward convergence stability, and low path planning efficiency of traditional deep reinforcement learning algorithms, this paper proposes a BiLSTM-D3QN (Bidirectional Long and Short-Term Memory Dueling Double Deep Q-Network) path planning algorithm based on the DDQN (Double Deep Q-Network) decision model. Firstly, a Bidirectional Long Short-Term Memory network (BiLSTM) is introduced to make the network have memory, increase the stability of decision making and make the reward converge more stably; secondly, Dueling Network is introduced to further solve the problem of overestimating the Q-value of the neural network, which makes the network able to be updated quickly; Adaptive reprioritization based on the frequency penalty function is proposed. Experience Playback, which extracts important and fresh data from the experience pool to accelerate the convergence of the neural network; finally, an adaptive action selection mechanism is introduced to further optimize the action exploration. Simulation experiments show that the BiLSTM-D3QN path planning algorithm outperforms the traditional Deep Reinforcement Learning algorithm in terms of network convergence speed, planning efficiency, stability of reward convergence, and success rate in simple environments; in complex environments, the path length of BiLSTM-D3QN is 20 m shorter than that of the improved ERDDQN (Experience Replay Double Deep Q-Network) algorithm, the number of turning points is 7 fewer, the planning time is 0.54 s shorter, and the success rate is 10.4% higher. The superiority of the BiLSTM-D3QN algorithm in terms of network convergence speed and path planning performance is demonstrated.

## 1 Introduction

In recent years, there has been a notable increase in the utilization of mobile robots in a variety of fields, including the military and industry, for the performance of essential unmanned tasks (Deguale et al., 2024). As the complexity of use scenarios and the level of safety standards continue to evolve, the challenge of efficiently and accurately planning driving paths has become a significant research topic (Meng et al., 2023). The planning of robot paths has consistently been a topic of significant interest within the field of robotics. It enhances the autonomy and operational efficiency of robots in diverse environments, offering substantial benefits for applications across various sectors of modern society (Kong et al., 2024). The fundamental objective of path planning is to identify the optimal route from the initial position to the intended destination of the robot, which typically necessitates a comprehensive assessment of factors such as distance, time, energy consumption, and the safety of the path (Guo et al., 2020). An optimal path should be free of obstacles, minimize detours, and facilitate efficient navigation (Junli et al., 2020). Mobile robots frequently operate in environments with diverse obstacles and unknown variables, underscoring the importance of path planning algorithms that can effectively avoid obstacles and operate in real-time with high reliability (Sha et al., 2023).

The existing body of literature on path planning algorithms can be divided into two main categories: traditional algorithms and those based on machine learning (Yuwan et al., 2022). The traditional path planning algorithms include Ant Colony Optimization (Lei et al., 2023), A* algorithm (Yu et al., 2024), Particle Swarm Optimization (Meetu et al., 2022), Dijkstra algorithm (Zhou et al., 2023), Genetic Algorithm (Debnath et al., 2024), Dynamic Window Approach (Chuanbo et al., 2023), and Artificial Potential Field algorithm (Zhang et al., 2023), among others. While these algorithms are widely used, they also have corresponding shortcomings, including low planning efficiency, poor search ability, and the tendency to fall into local optima. In recent years, the application of Deep Reinforcement Learning (DRL) algorithms in the field of path planning has been significantly advanced by the progress of hardware technology. DRL integrates the robust feature extraction capabilities of Deep Learning (DL) with the decision optimization abilities of Reinforcement Learning (RL), thereby offering a novel approach to address the limitations of traditional path planning algorithms (Chen et al., 2024). The DRL algorithm most widely used in path planning is the Deep Q Network (DQN) algorithm proposed by the DeepMind team (Lin and Wen, 2023). The DQN effectively addresses the “curse of dimensionality” problem faced by Q-value tables in complex environments by utilizing a neural network to replace the Q-value table in the Q-Learning algorithm. However, the traditional DQN algorithm exhibits shortcomings such as overestimation of Q-values and slow network convergence.

In light of the shortcomings of the DQN algorithm, Huiyan et al. (2023) put forth an augmented DDQN (double DQN) approach to path planning, which enhances the efficacy of algorithmic training and the precision of optimal path generation. Jinduo et al. (2022) put forth a double DQN-state splitting Q network (DDQNSSQN) algorithm that integrates state splitting with optimal states. This method employs a multi-dimensional state classification and storage system, coupled with targeted training to obtain optimal path information. Yan et al. (2023) put forth an end-to-end local path planner n-step dueling double DQN with reward-based *ϵ*-greedy (RND3QN) based on a deep reinforcement learning framework, which acquires environmental data from LiDAR as input and uses a neural network to fit Q-values to output the corresponding discrete actions. The problem of unstable mobile robot action selection due to sparse rewards is effectively solved. Shen and Zhao (2023) put forth a DDQN-based path planning framework for UAVs to traverse unknown terrain, which effectively mitigates the issue of overestimation of Q values. Wang et al. (2024) put forth an extended double deep Q network (DDQN) model that incorporates a radio prediction network to generate a UAV trajectory and anticipate the accumulated reward value resulting from action selection. This approach enhances the network’s learning efficiency. In a further development of the field, Li and Geng (2023) proposed a probabilistic state exploration ERDDQN algorithm. This has the effect of reducing the number of times the robot enters a repeated state during training, thus allowing it to explore new states more effectively, improve the speed of network convergence, and optimize the path planning effect. Tang et al. (2024) put forth a competitive architecture dueling-deep Q network (D3QN) for UAV path planning, which further optimizes the calculation of Q value and facilitates more precise updates to network parameters. Yuan et al. (2023) proposed the D3QN-PER path planning algorithm, which employs the Prioritized Experience Replay (PER) mechanism to enhance the utilization rate of crucial samples and accelerate the convergence of the neural network.

The aforementioned research has enhanced the functionality of the DQN algorithm to a certain degree; nevertheless, there are still significant issues that require attention, including low sample utilization, slow network convergence, and unstable reward convergence. To address this issue, this paper proposes a BiLSTM-D3QN path planning algorithm based on the DDQN algorithm. First, a BILSTM network is introduced to render the neural network memory-based, thereby increasing the stability of decision-making and thus facilitating more stable reward convergence. Secondly, a competitive network is introduced to further address the issue of overestimation of the neural network’s Q-value, thereby enabling the network to update more rapidly. The proposal of adaptive reprioritization of experience replay based on frequency penalty function is intended to facilitate the extraction of crucial and recent data from the experience pool, thus accelerating the convergence of the neural network. Finally, an adaptive action selection mechanism is introduced with the objective of further optimizing action exploration.

## 2 Related work

### 2.1 Q-Learning algorithm

**Figure 1:**
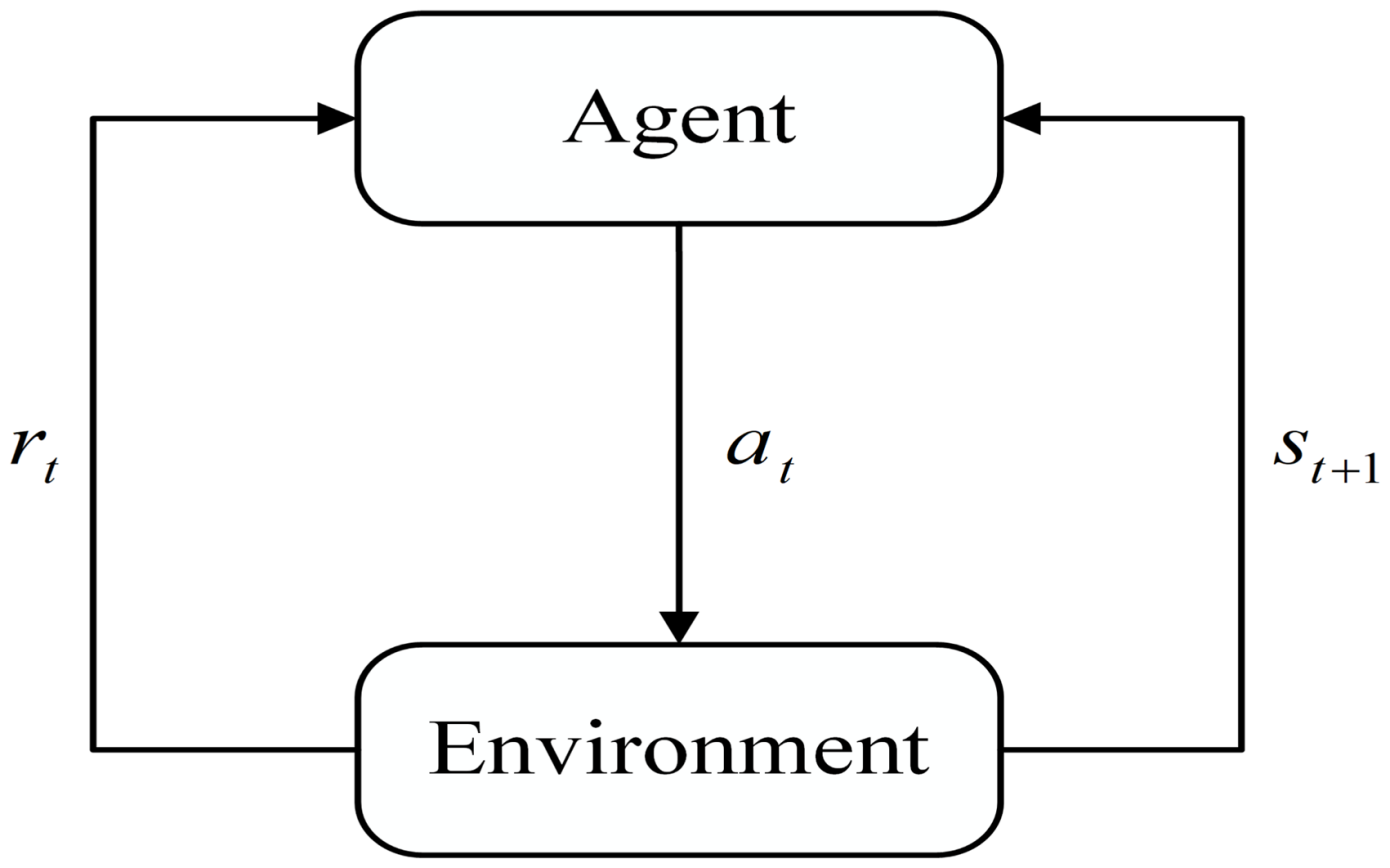
Basic reinforcement learning architecture.

Reinforcement Learning is an important branch of machine learning that aims to learn how to take the best action in a given situation by interacting with the environment in order to maximize cumulative rewards. Reinforcement learning differs from supervised learning and unsupervised learning in that it emphasizes trial and error in interacting with the environment for feedback, constantly adjusting strategies to achieve the best results. The basic framework of reinforcement learning is shown in Figure 1. The agent chooses action $a_{t}$ based on current state $s_{t}$. The environment provides reward $r_{t}$ for the current action and state $s_{t+1}$ for the next moment based on the action. After continuous interaction, the agent’s decision is improved and updated to obtain a higher reward $r_{t}$.

The Q-Learning algorithm is a model-free reinforcement learning method based on a value function. It selects the optimal strategy by updating the action value function Q. The update of Q value is shown in Equation 1:


(1) $$Q\left(s_{t},a_{t}\right)\leftarrow Q\left(s_{t},a_{t}\right)+\alpha \left[r_{t}+\gamma \max_{a^{\prime }}Q\left(s_{t+1},a^{\prime }\right)-Q\left(s_{t},a_{t}\right)\right]$$

Where $Q\left(s_{t},a_{t}\right)$ denotes the Q-value of the intelligent body for selecting action $a_{t}$ in the current state $s_{t}$; α denotes the learning rate; γ denotes the discount factor; $r_{t}$ denotes the reward value obtained after executing action $a_{t}$, and $\max_{a^{\prime }}Q\left(s_{t+1},a^{\prime }\right)$ denotes the maximum Q-value for the next state.

### 2.2 DQN algorithm

**Figure 2:**
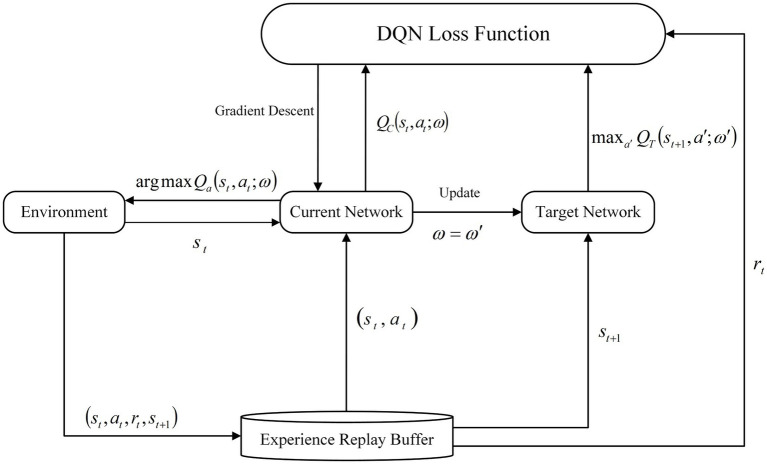
Schematic diagram of DQN structure.

The traditional DQN algorithm, as proposed by the DeepMind team, is based on the Q-Learning algorithm. The introduction of a neural network as the carrier of the value function allows for the nonlinear approximation of the state value function through the use of a neural network with parameters ω and an activation function. This approach enhances the efficiency of path planning. In contrast to the Q-Learning algorithm Q-value table, the DQN employs a neural network to address the dimensional explosion problem that arises in complex environments (Chen et al., 2024). However, the conventional DQN approach selects the action with the maximum Q-value when searching for the optimal action, which is susceptible to overestimation of the Q-value during network updates. Figure 2 illustrates the structure of the DQN algorithm.

The DQN neural network comprises two networks with identical structures: the current network ($Q_{C}$) and the target network ($Q_{T}$). The algorithm employs the current network to calculate an estimated value for a given state and utilizes the output value of the target network in conjunction with a sequential difference method to perform gradient descent, thereby updating the current network. Once the current network has undergone a specified number of updates, the target network is updated by copying the parameters C of the current network. During the training phase, a random and uniform sample is selected from the experience replay pool and provided to the two neural networks for the purpose of gradient descent with respect to the loss function. The calculation formula is presented in Equations 2,3.


(2) $$L_{\mathrm{Loss}}={\left(Q_{C}\left(s_{t},a_{t};\omega \right)-Q_{t\arg et}\right)}^{2}$$

(3) $$Q_{t\arg et}=r_{t}+\gamma \max_{a^{\prime }}Q_{T}\left(s_{t+1},a^{\prime };\omega^{\prime }\right)$$

Where ω is the current network parameter and $\omega^{\prime }$ is the target network parameter; γ is the discount factor; $Q_{C}\left(s_{t},a_{t};\omega \right)$ is the current network output value; and $\max_{a^{\prime }}Q_{T}\left(s_{t+1},a^{\prime };\omega^{\prime }\right)$ is the maximum action value of the target network at state $s_{t+1}$.

Following the calculation of the loss value, the DQN updates the network parameter ω through the application of the gradient descent method. The gradient descent formula is presented in Equation 4.


(4) $$\omega_{t+1}=\omega_{t}+E\left[Q_{t\arg et}-Q_{C}\left(s_{t},a_{t},\omega_{t}\right)\right]\nabla Q_{C}\left(s_{t},a_{t},\omega_{t}\right)$$

### 2.3 DDQN algorithm

**Figure 3:**
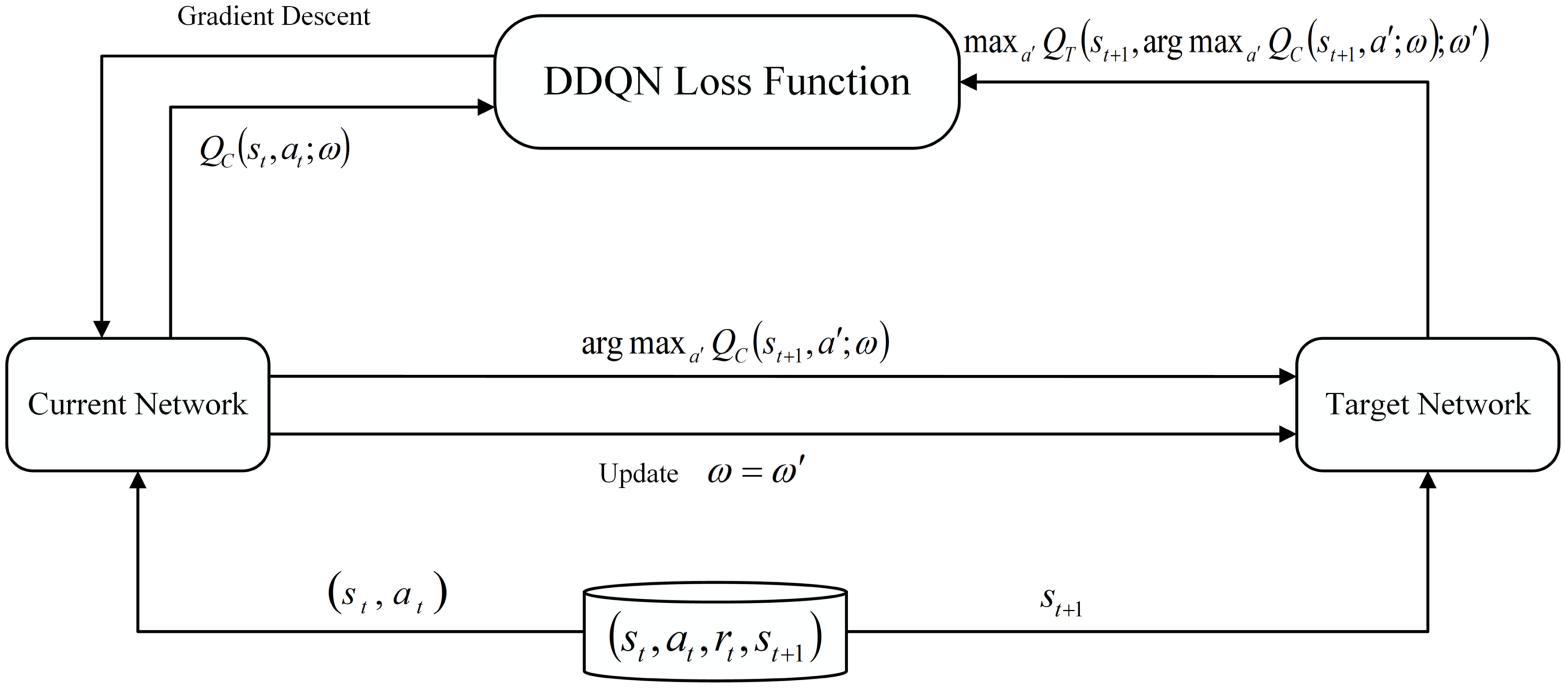
Schematic diagram of DDQN structure.

In response to the issue of overestimation of the Q value in the DQN algorithm, the DeepMind team put forth the DDQN algorithm as a potential solution. In comparison to the DQN algorithm, the DDQN algorithm modifies the manner in which the Q value is calculated within the target network. This involves the decomposition of the maximization operation within the target network into the utilization of distinct networks for the purposes of action selection and action evaluation. In contrast to the conventional approach of selecting the maximum Q value, the DDQN algorithm initially identifies the action $a^{\prime }$ that corresponds to the maximum Q value in state $s_{t+1}$ through the current network. The target network then calculates the Q value based on action $a^{\prime }$ and the current state $s_{t+1}$. This process effectively mitigates the issue of overestimation of Q values (Yuan et al., 2023), leading to more precise Q value estimation. The structural diagram of the DDQN network is illustrated in Figure 3.

The Target Value in the DDQN algorithm is calculated as shown in Equation 5:


(5) $$Q_{t\arg et}=r_{t+1}+\gamma Q_{T}\left(s_{t+1},\arg\max_{a^{\prime }}Q_{C}\left(s_{t+1},a^{\prime };\omega \right);\omega^{\prime }\right)$$

where $a^{\prime }$ represents the set of all possible actions in the next state $s_{t+1}$; and $\arg\max_{a^{\prime }}Q_{C}\left(s_{t+1},a^{\prime };\omega \right)$ represents the action with the largest Q value selected by the current Q network in $s_{t+1}$.

## 3 BiLSTM-D3QN path planning algorithm

While DDQN addresses the issue of overestimation of Q-values to a degree, it nevertheless exhibits certain shortcomings and constraints. In the DDQN algorithm, the value of *ε* is a constant. In the latter stages of path planning, the robot may fail to identify the optimal path due to the random selection of actions. While the experience replay buffer addresses the issue of data correlation, it also presents a challenge in efficiently sampling representative experiences from the experience pool to accelerate network convergence. Furthermore, when the robot encounters the same obstacle, it may execute disparate actions, which impedes the value function from attaining convergence. This ultimately results in an unstable decision-making process. In light of these considerations, this paper puts forth a BiLSTM-D3QN path planning algorithm founded upon the DDQN decision-making model.

### 3.1 Design of reward function

In the context of mobile robot path planning, the term “state” is defined as the position coordinates of the robot, as illustrated in Equation 6:

(6) $$S=\left(x,y\right)$$

**Figure 4:**
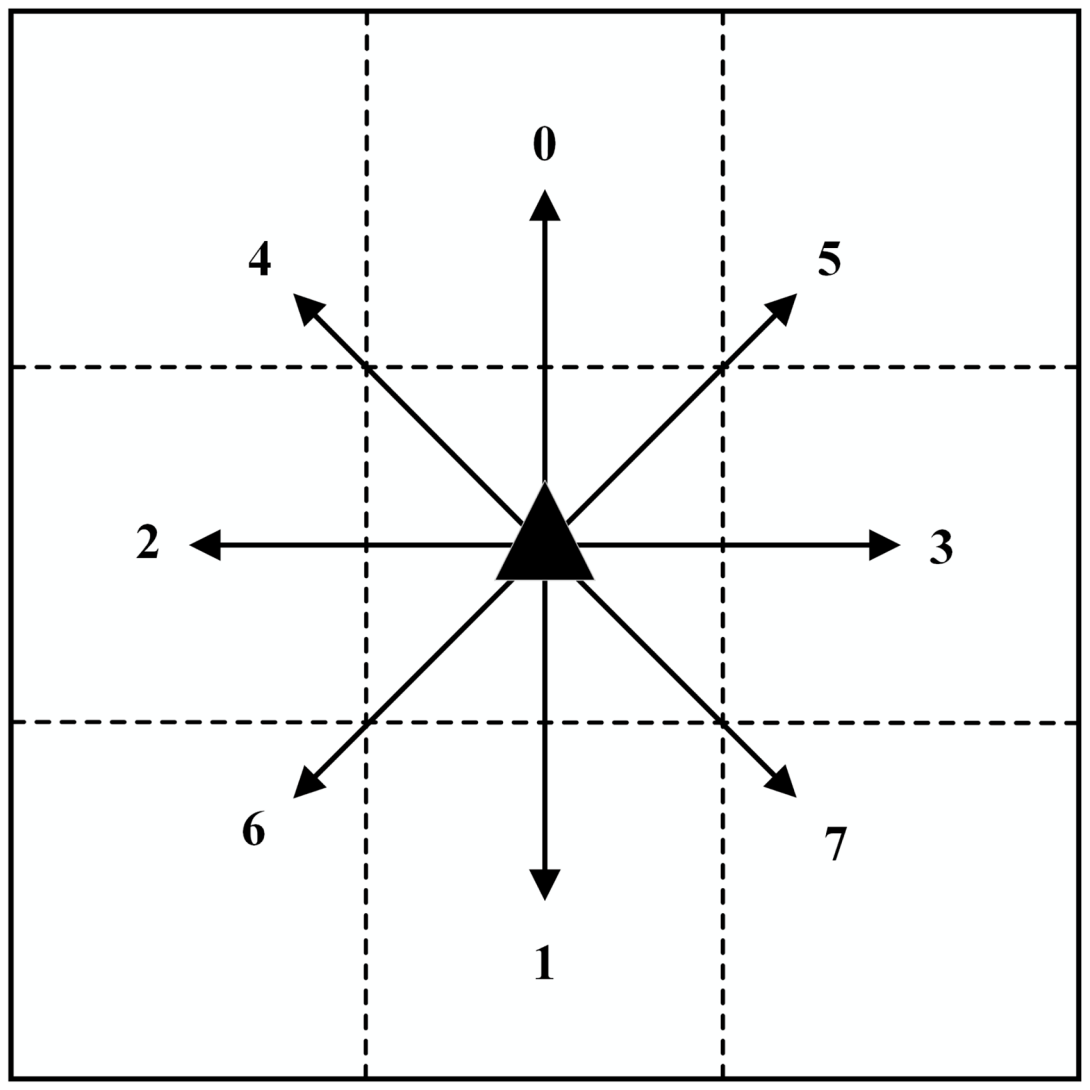
Schematic diagram of the action space.

Its action space is shown in Figure 4.

The area in which the robot is permitted to move is divided into a grid, and the robot is therefore able to move freely to any of the eight surrounding grids. The digits 0 through 7 are used to represent the eight directions: front, back, left, right, top left, top right, bottom left, and bottom right, respectively. The action set is illustrated in Equation 7:

(7) $$A=\left(0,1,2,3,4,5,6,7\right)$$

Reward functions are a key component of research in the field of RL on path planning. Classical DRL algorithms typically employ sparse reward functions, as discussed in Zhao et al. (2024). The classical DRL algorithm is designed with arrival rewards and collision rewards, with a positive reward of 20 given for arrival at the target point and-20 for collision, and the reward function is shown in Equation 8:

(8) $$r_{t}=\{\;\begin{array}{l} 20\;\mathrm{reach}\;\mathrm{goal}\\ -20\;\mathrm{collision} \end{array}$$

Since only the arrival and collision rewards are set during the training process, it results in a sparse reward signal. When the mobile robot acts, ineffective actions often occur. In general, terms, when the robot completes the action, if the reward value is 0 at this time, the robot can not judge what to do next based on the current state, and it is not clear how to reach the target position. Due to the difficulty of pre-exploration, the robot needs to go through a longer trial-and-error process to find the correct path. During this process, the robot is mainly guided by the negative rewards from collisions and lacks the guidance of positive rewards, so it is difficult to update a better strategy during strategy evaluation.

One solution to the reward sparsity problem is to add auxiliary rewards, and in this paper, we introduce a dynamic reward function. Distance and direction rewards are added to the environment as auxiliary rewards, and the reward values are dynamically presented with the change of robot position. The closer the mobile robot is to the target point, the greater the reward is. The reward function is shown in Equation 9:


(9) $$r_{t}=\{\;\begin{array}{ll} 20 & \;\mathrm{reach}\;\mathrm{goal}\\ \frac{j}{{\left(\Delta l\right)}^{3}} & \;\mathrm{other}\\ -2 & \;a\in A\left(0,1,2,3\right)\\ -2.5 & \;a\in A\left(4,5,6,7\right)\\ -5 & \;\mathrm{out}\;\mathrm{of}\;\mathrm{step}\\ -20 & \;\mathrm{collision} \end{array}$$

To ensure safety in the path planning process, the simulation environment is given to the obstacle expansion. When the distance between robot and the obstacle is less than 0.1 m it is considered to have collision, and the distance between the robot and the target point is less than 0.1 m it is considered to have reached the target point, and the distance between the mobile robot and the target point is calculated as shown in Equation 10:


(10) $$\Delta l=\sqrt{{\left(x_{\mathrm{current}}-x_{\mathrm{goal}}\right)}^{2}+{\left(y_{\mathrm{current}}-y_{\mathrm{goal}}\right)}^{2}}$$

where Δl is the Euclidean distance between the robot and the target position at time t; j is the distance-assisted reward constant, which is used to adjust the scale of the reward; $\left(x_{\mathrm{current}},y_{\mathrm{current}}\right)$ is the current position of the mobile robot and $\left(x_{\mathrm{goal}},y_{\mathrm{goal}}\right)$ is the target point position.

The improved dynamic reward function in this paper is shown in Equation 11:


(11) $$r_{t}=\frac{j}{{\left(\Delta l\right)}^{3}}$$

In the gradient update of the neural network value function, the error term is shown in Equation 12:


(12) $$\delta_{t}=Q_{t\arg et}-Q_{C}\left(s_{t},a_{t},\omega_{t}\right)$$

When the reward $r_{t}$ is sparse, the change of $\delta_{t}$ is drastic and has high variance, which affects the convergence. By introducing a smooth reward function, the variance of the gradient update term and the gradient update formula are shown in Equations 13 and 14:


(13) $$Var\left(\delta_{t}\right)=Var\left(Q_{t\arg et}\right)+Var\left(Q_{C}\left(s_{t},a_{t},\omega_{t}\right)\right)$$

(14) $$\nabla_{\omega }L_{\mathrm{Loss}}\left(\omega \right)=E\left[Q_{t\arg et}-Q_{C}\left(s_{t},a_{t},\omega_{t}\right)\right]\nabla Q_{C}\left(s_{t},a_{t},\omega_{t}\right)$$

Sparse rewards can lead to the following problems:

1. The target value $Q_{t\arg et}$ is discontinuous: when the rewards are sparse, $r_{t}$ is zero in most time steps, resulting in an unsmooth change in the value of $Q_{t\arg et}$;
2. Invalid gradient: When $r_{t}$ is mostly zero, the update signal ($Q_{t\arg et}-Q_{C}\left(s_{t},a_{t},\omega_{t}\right)$) of the gradient becomes sparse and has high variance, leading to difficulty in optimization. By introducing a smooth reward function with continuous non-zero values, the problem of sparse reward where most of $r_{t}$ is zero is eliminated. The continuity of $r_{t}$ makes the distribution of the objective value $Q_{t\arg et}$ more continuous, which directly reduces the variance of $Q_{t\arg et}$. Convergence is improved.

**Figure 5:**
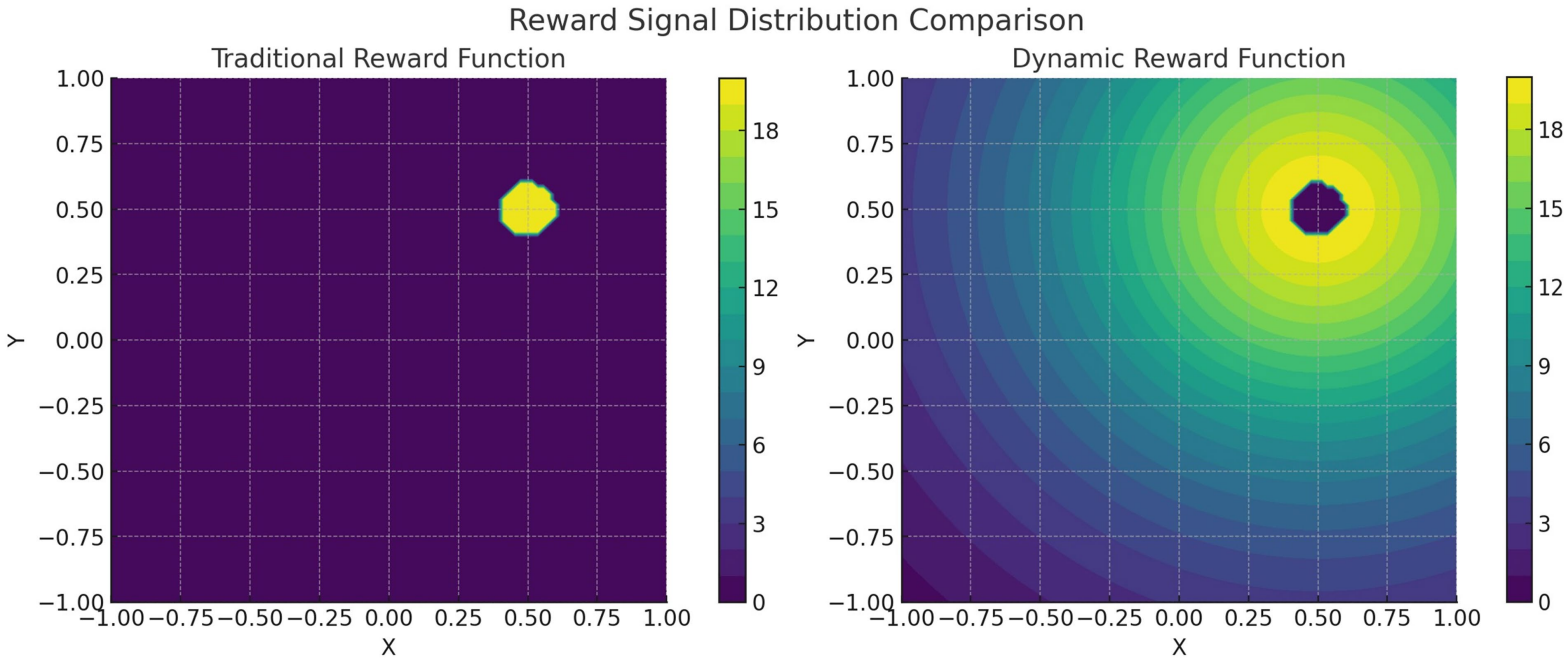
Visualization of signal distributions with traditional and dynamic reward functions.

The visualization of the signal distribution for the traditional and dynamic reward functions is shown in Figure 5. Assuming that the target point location of the mobile robot is (0.50, 0.50), the highlighted reward peaks at 20 when the Euclidean distance Δl between the robot and the target location is a minimum value of 0.1. The traditional reward function reward signal on the left side is only activated in a small area (with a radius of about 0.1) of the target point, and the other regions are almost unrewarded. The reward signal is very sparse, leading to inefficient training of the reinforcement learning algorithm. The right dynamic reward function reward signal gradually increases as the distance between the mobile robot and the target point decreases, and the color gradually changes from bright (high reward value) to dark (low reward value), forming a smooth gradient field. The dynamic reward function can alleviate the sparse reward problem by extending the reward coverage and establishing a gradient reward field, thus significantly improving the algorithm’s performance. This gradient reward design can effectively guide the mobile robot towards the target direction.

### 3.2 Adaptive action selection mechanism

In the traditional action selection strategy, *ε* in the ε-greedy strategy is a fixed value. First, this may lead to insufficient exploration in the early stage of the system, which is easy to fall into the local optimal solution and unable to find the global optimal solution. In addition, there is too much exploration in the later stage, which can lead to slower convergence and the inability to effectively utilize the currently learned optimal policy, affecting the final performance. To avoid falling into a local optimum, the ε-greedy policy is improved so that the value of ε is no longer a fixed value and decreases linearly as planning progresses. The action selection mechanism in the early stage of planning selects actions more randomly by probability, and in the later stage it is more likely to select actions with large reward values. The action selection function is shown in Equation 15:


(15) $$a_{t}=\left\{\begin{array}{ll} \arg\max_{a}Q\left(s_{t},a;\omega \right) & n>\varepsilon \\ \mathrm{random}\left(A\;\right) & n\leq \varepsilon \end{array}\right.$$

where n is a random number between 0 and 1; the exploration factor ε represents the degree of random exploration of the environment; and the set of all actions is denoted A.

At each time step t, the selection of action $a_{t}$ is divided into two cases:

1. When n is greater than ε, the action a that maximizes $Q\left(s_{t},;a,;\omega \right)$ in the current state $s_{t}$ is selected. This means that in this case the algorithm prefers to select the action with the highest reward in the current estimation, i.e., it utilizes the best-known action.
2. When n is less than ε, choose a random action from the set of all possible actions A. This means that in this case, the algorithm prefers to randomly explore new actions to discover potentially better strategies.

ε dynamic adjustment is shown in Equation 16:


(16) $$\varepsilon_{t}=\varepsilon_{\text{min}}+\left(\varepsilon_{\text{max}}-\varepsilon_{\text{min}}\right)\cdot \left(1-\frac{t}{T}\right)$$

where t is the current number of cycles; T is the total number of cycles; $\varepsilon_{\text{max}}$ is the maximum exploration rate; and $\varepsilon_{\text{min}}$ is the minimum exploration rate.

To achieve a smooth transition of ε, the most commonly used method is linear decay. As the number of cycles of t increases, the value of $\left(1-\frac{t}{T}\right)$ gradually decreases, and the exploration rate ε gradually decreases from the maximum value $\varepsilon_{\text{max}}$ to the minimum value $\varepsilon_{\text{min}}$. When t is equal to 0, $\varepsilon_{t}$ is equal to $\varepsilon_{\text{max}}$, which means that in the initial stage, the exploration factor is at the maximum value, and the algorithm prefers random exploration. When t is greater than 0 and less than T, the exploration factor gradually finds a balance between exploration and utilization. When t is equal to T, $\varepsilon_{t}$ is equal to $\varepsilon_{\text{min}}$, which means that in the final stage, the exploration factor is at its minimum value and the algorithm prefers to utilize the best-known action. This satisfies the exploration degree in the early stage and avoids missing the optimal path in the later stage, while retaining the possibility of randomly selecting actions with lower probability.

### 3.3 Adaptive reprioritization of experience replay based on frequency penalty function

In traditional DQN algorithms, experience playback is usually done by sampling uniformly from the experience pool, which is less efficient. Therefore, academics have proposed a prioritized experience replay (PER) based on temporal difference error (TD Error), where experiences with large TD Error usually indicate higher learning value. However, there are places where PER can be optimized. In this paper, we propose adaptive reprioritization of experience replay based on frequency penalty function, whose core concept is to reflect the change in importance of experience by combining the TD Error and the frequency with which the experience is used. The frequency-of-use-based penalty function reduces the probability of being sampled again by dynamically adjusting the priority of those experiences that have been sampled multiple times. The penalization function using the frequency and the prioritization of experience are shown in Equations 17 and 18:


(17) $$f\left(u_{i}\right)=\frac{1}{1+\mu u_{i}}$$

(18) $$p_{i}={\left(\delta_{i}+\rho \right)}^{\nu }\cdot f\left(u_{i}\right)$$

where $p_{i}$ is the priority of data i; $\delta_{i}$ is the TD Error; the parameter ρ is set to avoid $p_{i}$ being 0; ν is a parameter controlling the degree of amplification of the priority; $f\left(u_{i}\right)$ is the penalization function using the frequency; $u_{i}$ is the number of times the experience i has been sampled; and μ is the penalty rate constant.

The probability of each piece of data being drawn is shown in Equation 19:


(19) $$P\left(i\right)=\frac{p_{i}}{\sum_{k}p_{k}}$$

where the denominator is the sum of all data priorities; k is the number of data in the experience pool.

The preference for playing back experiences with high TD error leads to the problem that the neural network training process is prone to oscillations or even divergence. Therefore the importance of sampling weight $\omega_{i}$ is introduced to solve this problem. As shown in Equation 20:


(20) $$\omega_{i}={\left(\frac{1}{N}\cdot \frac{1}{P_{i}}\right)}^{\beta }$$

where N is the number of data in the experience pool; parameter β controls the influence of importance sampling weights in the learning process.

### 3.4 Dueling Network

**Figure 6:**
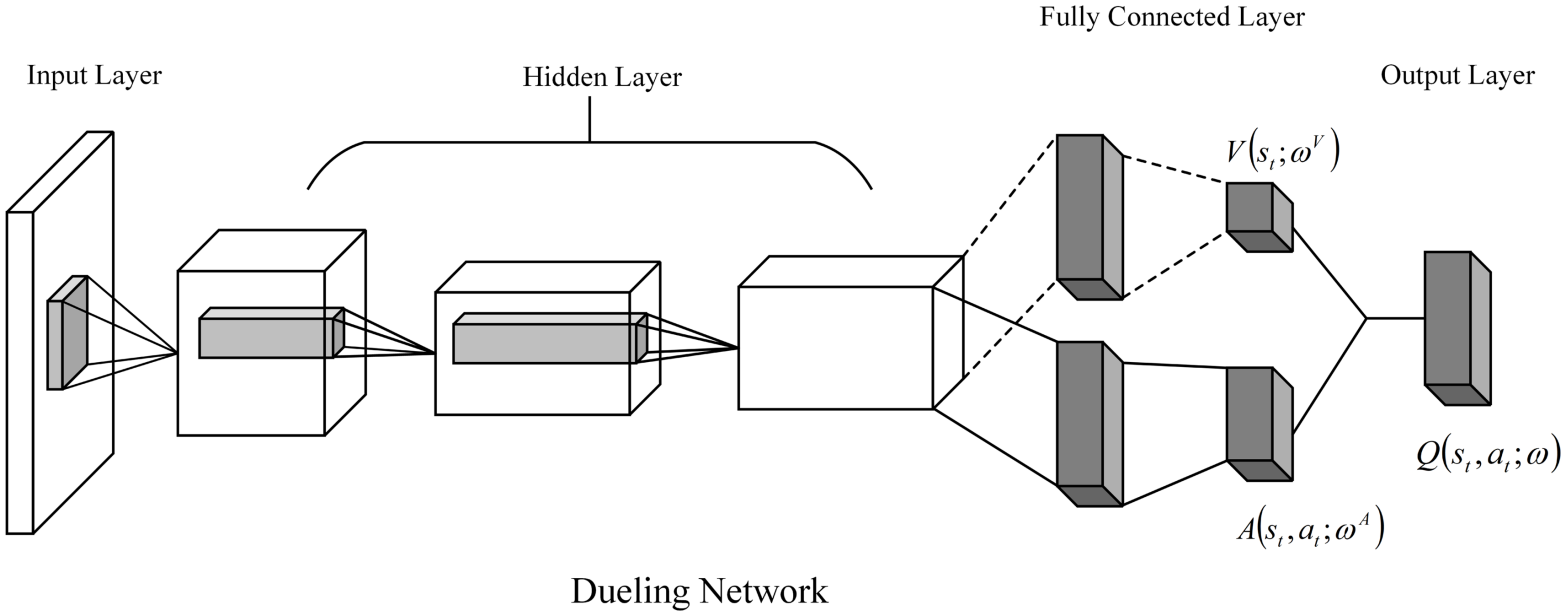
Schematic diagram of the Dueling Network architecture.

This paper introduces a competitive network into the neural network structure of the DDQN algorithm. The competitive network introduces a dual layer with two branches between the hidden layer and the output layer of the DDQN network, which are, respectively, the advantage function layer A and the state value function layer V. The advantage function layer calculates the advantage of each action relative to the average, and the state function layer calculates the state value of the object in its current state. The advantage value of each action is summed with the state value to obtain the Q-value of each action. The problem of overestimation of the Q-value by the neural network can be further solved. The structure of the competition network is shown in Figure 6.

The Q value calculation for the Dueling DQN is shown in Equation 21:


(21) $$Q\left(s_{t},a_{t};\omega \right)=A\left(s_{t},a_{t};\omega^{A}\right)+V\left(s_{t};\omega^{V}\right)-\frac{1}{\left|A\right|}\underset{a^{\prime }}{\sum }A\left(s_{t},a^{\prime };\omega^{A}\right)$$

where $A\left(s_{t},a_{t};\omega^{A}\right)$ is the dominant value function in state $s_{t}$; $V\left(s_{t};\omega^{V}\right)$ is the state value function in state $s_{t}$; $\omega^{A}$ and $\omega^{V}$ are the network parameters of the dominant and state value functions, respectively; |A| is the size of the action space; and $\underset{a^{\prime }}{\sum }A\left(s_{t},a^{\prime };\omega^{A}\right)$ is the average of all action values obtained from the dominant value function layer.

This is precisely because in a competitive network, the Q value of network Q is calculated by summing up the Value Function and the Advantage Function. The existence of the Value Function allows the Algorithmic Network to evaluate the state value that is not affected by actions, thereby improving the accuracy of the Q value calculation and the algorithm’s efficiency.

### 3.5 Bidirectional long short-term memory network

In this paper, a Bidirectional Long Short-Term Memory Network (BiLSTM) is introduced to solve the problem of poor decision-making due to partial observability based on the DDQN decision model. By adding BiLSTM, the action selection is correlated before and after. The decision-making is more stable when facing the same obstacle, and the reward converges more stably in path planning.

#### 3.5.1 Long short-term memory network

**Figure 7:**
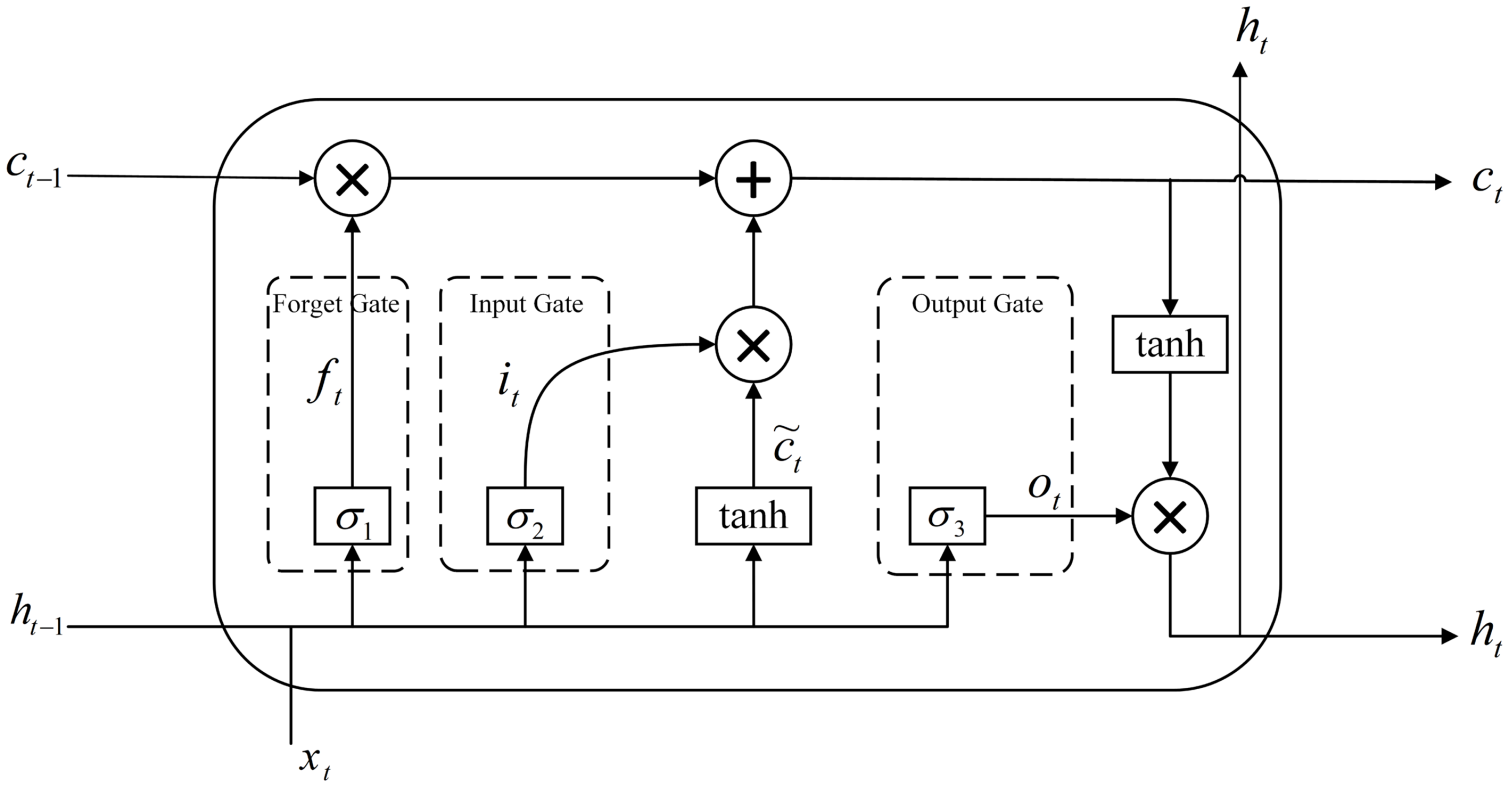
Schematic diagram of the LSTM structure.

Long Short-Term Memory (LSTM) is an improvement of Recurrent Neural Network (RNN), which solves the problem of “vanishing gradient” in model training by adding memory units. The basic units of the LSTM network include forgetting gates, input gates, and output gates. Therefore, LSTM can effectively retain and update long-term memory and process complex time series data. The network structure is shown in Figure 7.

$f_{t}$ is the output of the forgetting gate at the moment t; $i_{t}$ is the output of the input gate at the moment t; $o_{t}$ is the output of the output gate at the moment t; $c_{t}$ is the cellular state of the memory cell LSTM at the current moment; $c_{t-1}$ is the cellular state of the memory cell LSTM at the previous moment; $x_{t}$ is the input vector at the current moment; $h_{t}$ is the output vector at the current moment; σ is the activation function; and $h_{t-1}$ is the output at the previous moment.

The core of LSTM is the forgetting gate, which is responsible for preserving long-term memory and is used to decide the data to be preserved in the historical information and to select the memorized information for the Sigmoid nonlinear transformation. Its formula is shown in Equation 22:


(22) $$f_{t}=\sigma \left(W_{xf}x_{t}+W_{hf}h_{t-1}+b_{f}\right)$$

where $W_{xf}$ and $W_{hf}$ are the weight parameters of the forgetting gate; $b_{f}$ is the bias parameter of the forgetting gate.

The input gate is used to control the size of the data flowing into the memory cell, and the information in the memory cell can be updated using Equation 23 and Equation 24:


(23) $$i_{t}=\sigma \left(W_{\mathrm{xi}}x_{t}+W_{hi}h_{t-1}+b_{i}\right)$$

(24) $${\tilde{C}}_{t}=\tanh\left(W_{C}x_{t}+W_{C}h_{t-1}+b_{C}\right)$$

where $W_{\mathrm{xi}}$ and $W_{hi}$ are the weight matrices of the input gates; $b_{i}$ is the bias vector of the input gates; ${\tilde{C}}_{t}$ is the temporary variable used to compute $c_{t}$; $W_{C}$ is the neural network parameter; and $b_{C}$ is the deviation vector.

The effect of the previous moment memory cell on the current moment memory cell can be expressed by Equation 25:


(25) $$c_{t}=f_{t}⊙c_{t-1}+i_{t}⊙{\tilde{C}}_{t}$$

where ⊙ stands for the Hadamard product operation.

The output gate is used to determine the output value of the LSTM network and the current input features and the previous moment output are passed to the activation function to compute the output at the current moment. The computational formula is shown in Equation 26 and Equation 27:


(26) $$h_{t}=o_{t}⊙\tanh\left(c_{t}\right)$$

(27) $$o_{t}=\sigma \left(W_{xo}x_{t}+W_{ho}h_{t-1}+b_{o}\right)$$

where $W_{xo}$ and $W_{ho}$ are the weight parameters of the output gate; $b_{o}$ is the bias parameter of the input gate.

#### 3.5.2 Bidirectional long short-term memory network

**Figure 8:**
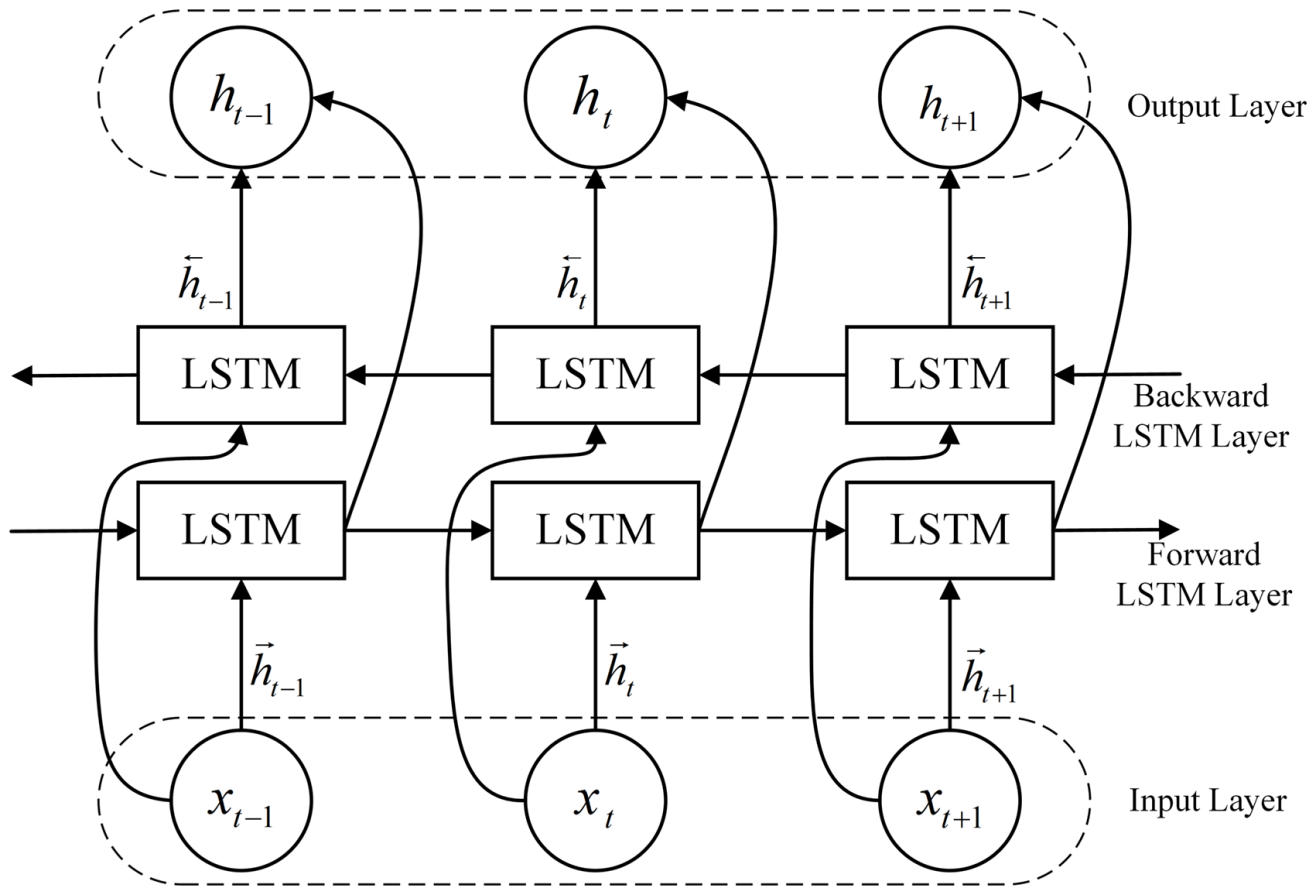
Schematic diagram of the BiLSTM structure.

BiLSTM Network is an improved LSTM network that considers both past and future information while processing sample data. The model combines a forward-backward LSTM layer and a backward-forward LSTM layer on top of a unidirectional LSTM network. This allows for more comprehensive use of information from the sample data in training, improved accuracy in the sequence labeling task, and a reduction in information loss, which increases the stability of decision-making. The structure is shown in Figure 8.

BiLSTM network is the original signal from the input layer into the network layer after the forward LSTM calculation to get an output value, the value will affect the learning rate and the output function, etc.; at the same time, after the reverse LSTM calculation to get the output value, which will determine the connection weights of the input nodes in the BiLSTM, to form a new set of output values. The calculation process is shown in Equations 28, 29:


(28) $$\vec{h}=\mathrm{LSTM}\left(x_{t},\vec{h_{t-1}}\right)$$

(29) $$\overset{\leftarrow }{h}=\mathrm{LSTM}\left(x_{t},\overset{\leftarrow }{h_{t-1}}\right)$$

where $x_{t}$ is the input at moment t, $\vec{h}$ is the output of the forward implicit layer at each moment, and $\overset{\leftarrow }{h}$ is the output of the backward implicit layer at each moment.

The outputs of the forward LSTM and the backward LSTM are merged at each time step, usually by splicing. The calculation formula is shown in Equation 30:


(30) $$h_{t}=\left[\vec{h_{t}}\overset{\leftarrow }{h_{t}}\right]$$

where || denotes vector concatenation.

### 3.6 BiLSTM-D3QN algorithm path planning overall process

**Figure 9:**
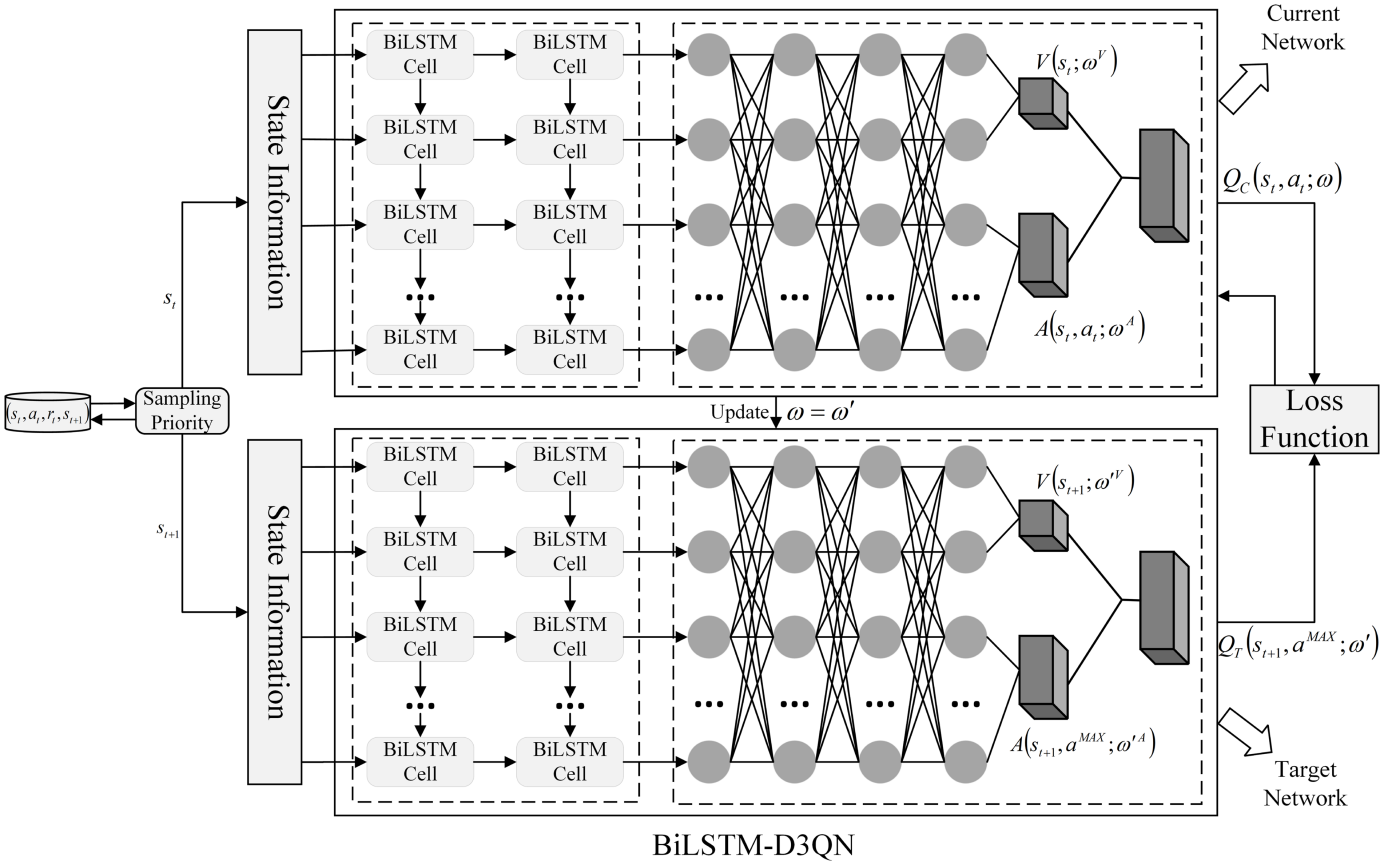
Overall framework of BiLSTM-D3QN algorithm.

The framework of the BiLSTM-D3QN model is shown in Figure 9. The BiLSTM-D3QN model adopts a dual network structure, which inputs the current state $s_{t}$ from the experience pool to the current network $Q_{C}$ based on the priority, and then selects the action $a_{t}$ under the state $s_{t}$ according to the adaptive action and obtains the Q-value of the current network. The state $s_{t+1}$ is input to the target network $Q_{T}$. At the same time, the action $a^{\mathrm{MAX}}$ corresponding to the maximum Q value in state $s_{t+1}$ is selected in the current network, and then $a^{\mathrm{MAX}}$ is used to find the Q value in the target network. The current network updates the network parameters ω by backpropagation of the loss function and periodically copies the parameters to the target network parameters $\omega^{\prime }$. In the inner layer of the two network structures, the state information is extracted through the memory unit module and input into the two-layer BiLSTM network. After passing through the four fully connected layers, the advantage function layer A and the state value function layer V output the Q value. This makes the mobile robot memorable and more stable in decision-making when encountering the same obstacles. The robot’s ability to find target points and avoid obstacles becomes stronger, which also makes the converged reward curve less volatile thus planning a better path. Path planning pseudocode of mobile robot based on BiLSTM-D3QN is shown in Algorithm 1.

#### ALGORITHM 1 Main program of BiLSTM-D3QN (Path planning pseudocode of mobile robot based on BiLSTM-D3QN)

1. Initialize an experience pool M with capacity N

2. Initialize learning rate, discount factor

3. The same parameters initialize the BiLSTM-current network $Q_{C}$ and the BiLSTM-target network $Q_{T}$

4. **for** episode =1 to T **do**

5.Initialize the environment

6.Initialize done = False

7.Initialize step counter = 0

8.Initialize accumulate reward = 0

9.**while not** done **and** step counter < max episode step **do**

10.Determine mobile robot state $s_{t}$

11.**if** mobile robots in an accessible state **then**

12.Selection of action $a_{t}$ based on probability ε

13.**otherwise** use a greedy strategy to select an action $a_{t}$

14.**end if**

15. Perform action $a_{t}$, get reward $r_{t}$, and new state $s_{t+1}$

16. Accumulate reward +=$r_{t}$

17. Store the experience data $\left(s_{t},a_{t},r_{t},s_{t+1}\right)$ in the experience pool M

18. Randomly select batch data samples $\left(s_{j},a_{j},r_{j},s_{j+1}\right)$ from M

19. **if** end of the episode **then**

20.Set $Q_{t\arg et}=r_{j}$

21. **else**

22.Set $Q_{t\arg et}=r_{t}+\gamma \max_{a^{\prime }}Q_{T}\left(s_{t+1},;a^{\prime },;\omega^{\prime }\right)$

23. **end if**

24. Perform gradient descent for network parameter ω in ${\left(Q_{C}\left(s_{t},;a_{t},;\omega \right)-Q_{t\arg et}\right)}^{2}$

25. Determine mobile robot state $s_{t+1}$

26.**if** mobile robot in an obstructed state **then**

27.Set done = False

28.Finish the episode

29.**else**

30.**continue**

31.**end if**

32.Step counter + = 1

33.**end while**

34. **end for**

## 4 Experimental results and analysis

### 4.1 Environment setup and parameter configuration

**Figure 10:**
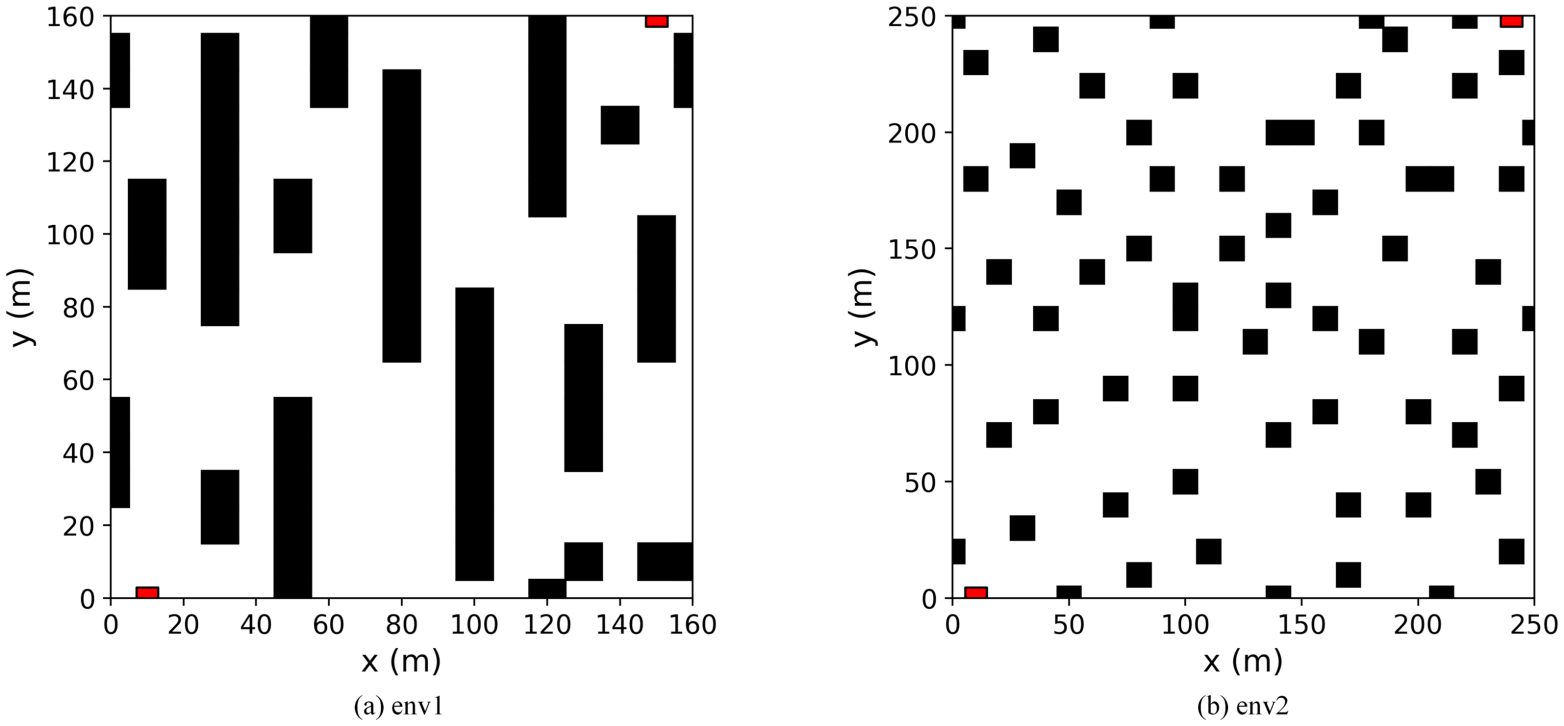
Map of the simulation environment. **(a)** Environment 1 is a simple environment. **(b)** Environment 2 is a complex environment.

A simulation comparison experiment was set up to verify the algorithm’s effectiveness proposed in this paper. Experimental environment: CPU model i7-13700H, GPU model RTX4070, Python 3.8, Pytorch 2.2.2, Tensorflow 2.13, Cuda 11.8. In this experiment, two raster environments were created. Environment 1 is a simple environment with a raster map size of 16 × 16; environment 2 is a complex environment with a raster map size of 25 × 25. In both environments, the black rectangular blocks represent obstacles, and the white parts are free-movement areas. The red square in the bottom left corner represents the starting point of the mobile robot; the red square in the top right corner represents the endpoint of the mobile robot. The starting point of Environment 1 is (10, 0) and the endpoint is (150, 160); the starting point of Environment 2 is (10, 0) and the endpoint is (240, 250). The robot can move on the map in eight directions: up, down, left, right, top left, top right, bottom left, and bottom right. Each movement is 10 m long. The episode ends when the robot hits an obstacle, exceeds the maximum number of steps, or reaches the end. The map of the simulation environment is shown in Figure 10.

1. Comparison with traditional DRL algorithms in a simple environment. The experimental environment is shown in environment 1 in Figure 10A. In Environment 1, the path planning of the Q-learning, DQN, DDQN, and the proposed BiLSTM-D3QN algorithm with the same parameters are performed, respectively. The superiority of the proposed algorithm over traditional DRL path planning algorithms in a simple environment is verified.
2. Comparison of the algorithm with improved algorithms in complex environments. The experimental environment is shown in environment 2 in Figure 10B. Q-learning, DQN, DDQN, and ERDDQN (Wang et al., 2024) with the same parameters are made to perform path planning with the BiLSTM-D3QN algorithm proposed in this paper, under Environment 2. Environment 2 is more complex than environment 1, and path planning is more difficult, which can better verify the effectiveness and robustness of the algorithm in this paper.

In both environments, 250 rounds of path planning are performed. The activation function used by the neural network is ReLU, the optimizer is Adam, and the network parameters are saved every 5 updates. The hyperparameter settings are shown in Table 1.

**Table 1:** The hyperparameter settings.

Parameters	Meaning	Value
α	Learning rate	0.001
γ	Discount factor	0.99
$\varepsilon_{\text{max}}$	Maximum exploration rate	0.6
$\varepsilon_{\text{min}}$	Minimum exploration rate	0.1
M	Replay memory capacity	100,000
B	Batch size	128
U	Update network at fixed interval	5
Step	Maximum steps	10,000
ν	Priority exponent	0.6
β	Importance sampling weight	0.4
μ	Penalty rate constant	0.01
j	Distance reward constant	0.1

### 4.2 Analysis of training results

#### 4.2.1 Comparison with traditional DRL algorithms in simple environment

**Figure 11:**
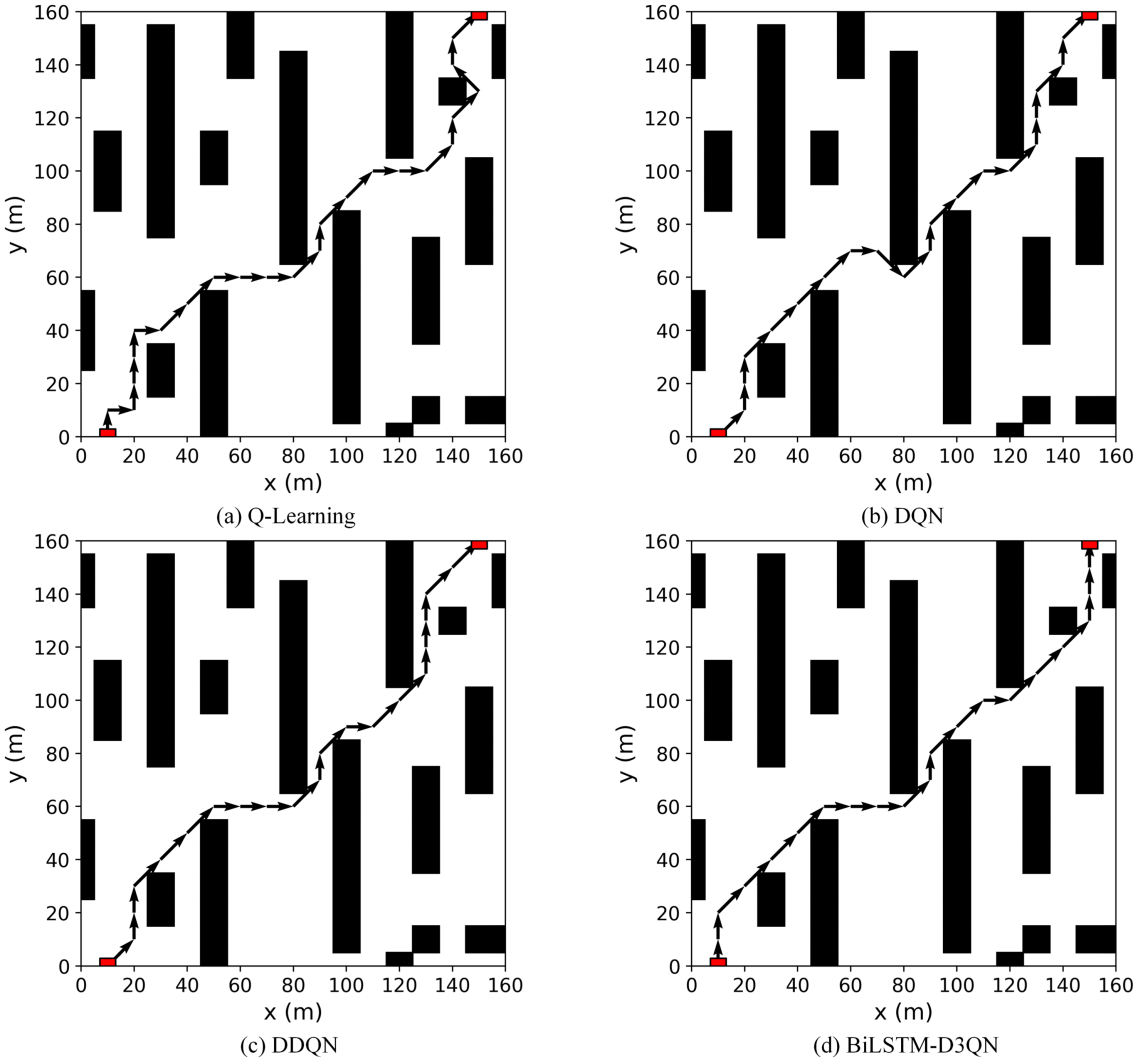
Comparison of the planned routes of the four algorithms in env 1. **(a)** Q-Learning algorithm; **(b)** DQN algorithm; **(c)** DDQN algorithm; (d) BilSTM-D3QN algorithm.

**Figure 12:**
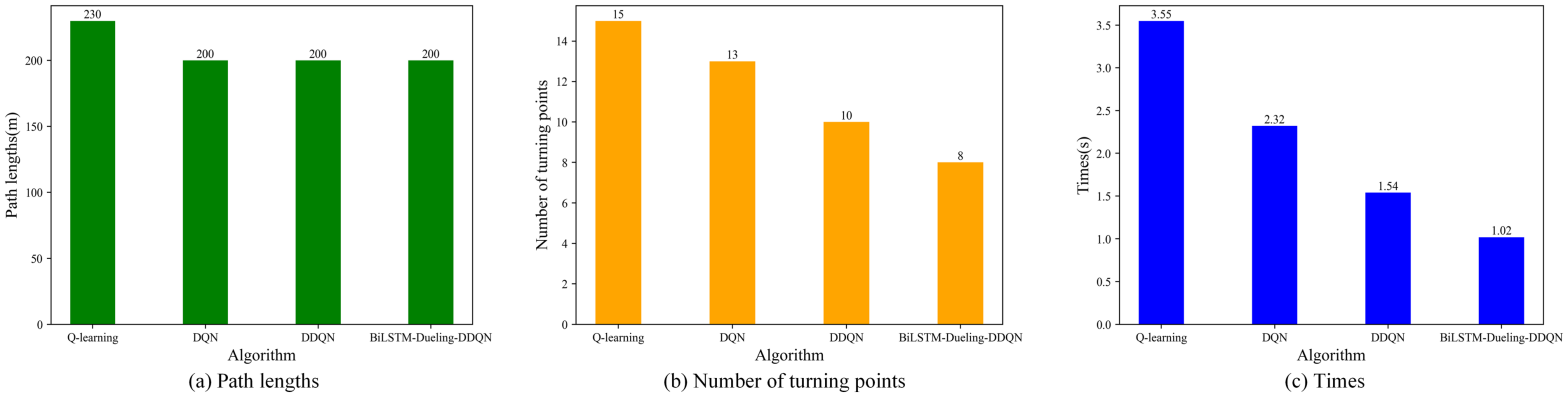
Comparison of the four algorithm indicators in env 1. **(a)** Path lengths; **(b)** Number of turning points; **(c)** Times.

To verify the effectiveness of the proposed algorithm, it is compared with the Q-learning, DQN, and DDQN path planning algorithms in environment 1. Figure 11 shows the path-planning route maps of the four algorithms, and Figure 12 compares the metrics of the four algorithms in environment 1, including the planned path length, the number of planned path turning points, and the time required for path planning. As can be seen from Figures 11, 12, the BiLSTM-D3QN algorithm plans an optimal path. Although the BiLSTM-D3QN algorithm and the DQN and DDQN algorithms both plan a path length of 200 m, the number of path turning points for the BiLSTM-D3QN algorithm is 8, which is lower than the 13 for the DQN algorithm and the 10 for the DDQN algorithm. The time required for path planning is 1.02 s, which is lower than the 2.32 s for the DQN algorithm and the 1.54 s for the DDQN algorithm. Of the four algorithms compared, the worst performer was the Q-learning algorithm. The path planned by the Q-learning algorithm was 230 m long, with 15 turning points and a planning time of 3.55 s. It can be seen that the BiLSTM-D3QN algorithm proposed in this paper is superior to the traditional Q-learning, DQN, and DDQN algorithms in terms of path planning.

From Table 2, it can be seen that in 250 training episodes, the algorithm with the highest overall success rate is the BiLSTM-D3QN algorithm proposed in this paper, which reaches 97.6%; followed by the DDQN algorithm 93.2% and the DQN algorithm 88.4%; while the overall success rate of the ordinary Q-learning algorithm is 80.4%. The BiLSTM-D3QN path planning algorithm has a greater advantage in the overall success rate. Meanwhile, from the point of view of the growth rate of the cumulative number of successes per 50 rounds, the average growth rate of the algorithm proposed in this paper is 0.995, which is higher than that of other algorithms, indicating that the neural network has the fastest convergence speed. Combined with the above analysis, the superiority of BiLSTM-D3QN is reflected in its faster growth rate of successful episodes and higher final success rate.

**Table 2:** Cumulative number of successes in env 1.

Episode	Q-learning	DQN	DDQN	BiLSTM-D3QN
50	34	36	41	45
100	71	77	86	94
150	113	123	134	144
200	159	171	183	194
250	208	221	233	244
Success rate	83.20%	88.40%	93.20%	97.60%

**Figure 13:**
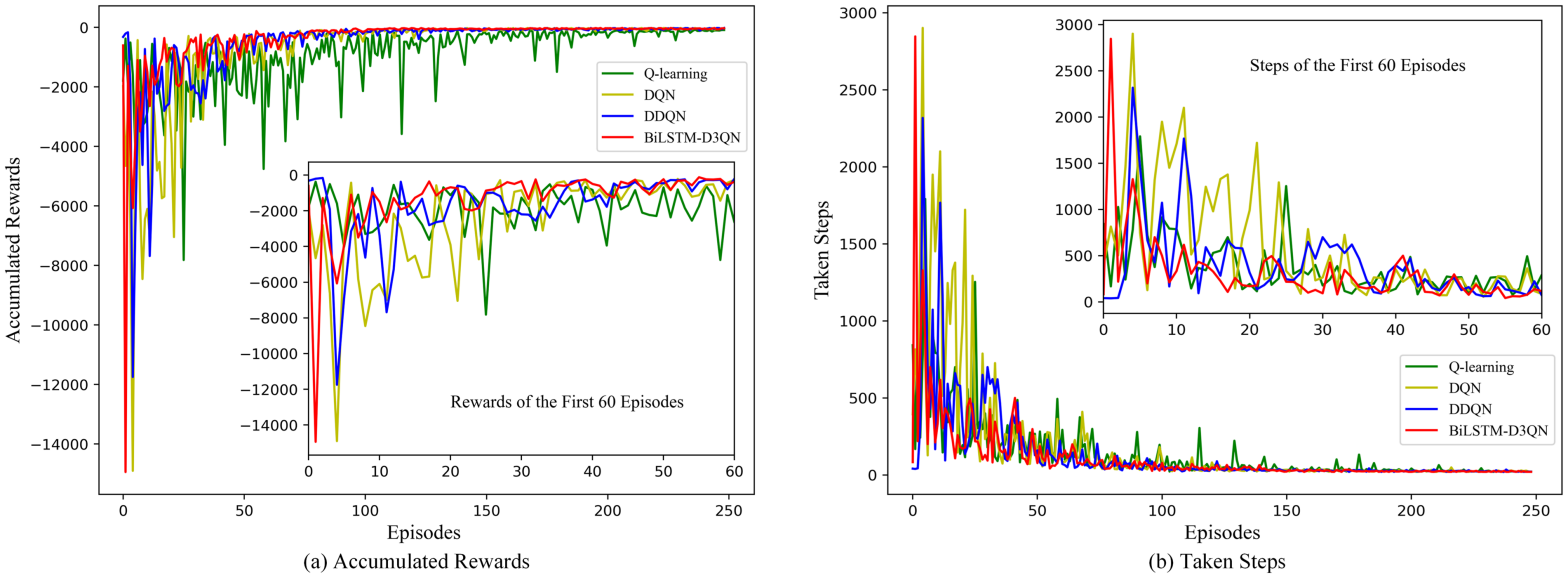
Comparison of rewards and steps accumulated per episode in env 1. **(a)** Accumulated rewards for each episode of path planning; **(b)** Taken steps for each episode of path planning.

Figure 13 shows a comparison of the cumulative reward and number of steps for each episode of path planning by the robot in Environment 1 for Q-learning, DQN, DDQN, and the proposed BiLSTM-D3QN algorithm. Green represents Q-learning, yellow represents DQN, blue represents DDQN, and red represents BiLSTM-D3QN. After 250 episodes of path planning in the same environment, all four algorithms were able to complete the robot’s path-planning task to the goal point, and the reward and step curves converged and eventually became similar. However, there were significant differences in the speed and stability of convergence. The Q-learning algorithm gradually converged after 120 episodes, and the curve fluctuated greatly after convergence. This indicates that during the later training process, although the mobile robot reaches the target point, it makes unstable decisions, causing the reward value to fluctuate greatly. The DQN and DDQN algorithms are actively explored during the first 40 training episodes. The reward curve and the step curve show fluctuations, and the DDQN algorithm is superior to the DQN algorithm in terms of accumulated reward value and number of steps in each episode. This is because the DDQN algorithm changes the way the Q-value of the target network is calculated, alleviating the problem of overestimating the Q-value in the DQN algorithm and making the network converge more stably. The BiLSTM-D3QN algorithm proposed in this paper converges rapidly after 10 episodes of exploration. After 30 episodes, the reward and step curves tend to stabilize and reach an optimal value, which is significantly faster than the other algorithms compared. This shows that the frequency penalty function of the algorithm in this paper reprioritizes the experience replay mechanism, which improves the use of important data and thus accelerates the convergence of the neural network. The final reward value exceeds that of DQN and DDQN, which is attributed to the fact that the competitive network in BiLSTM-D3QN has a separate data flow structure that can approximate the Q function more effectively and accurately. The algorithm in this paper has less variation in reward value in the later stages of training. The BiLSTM memory network relates the previous and subsequent actions so that the robot’s decisions are stable and the robot achieves a more stable cumulative reward during path planning. In summary, the BiLSTM-D3QN algorithm outperforms other algorithms during path planning training in terms of convergence speed, cumulative reward, and overall stability.

#### 4.2.2 Comparison with improved deep reinforcement learning algorithms in complex environment

**Figure 14:**
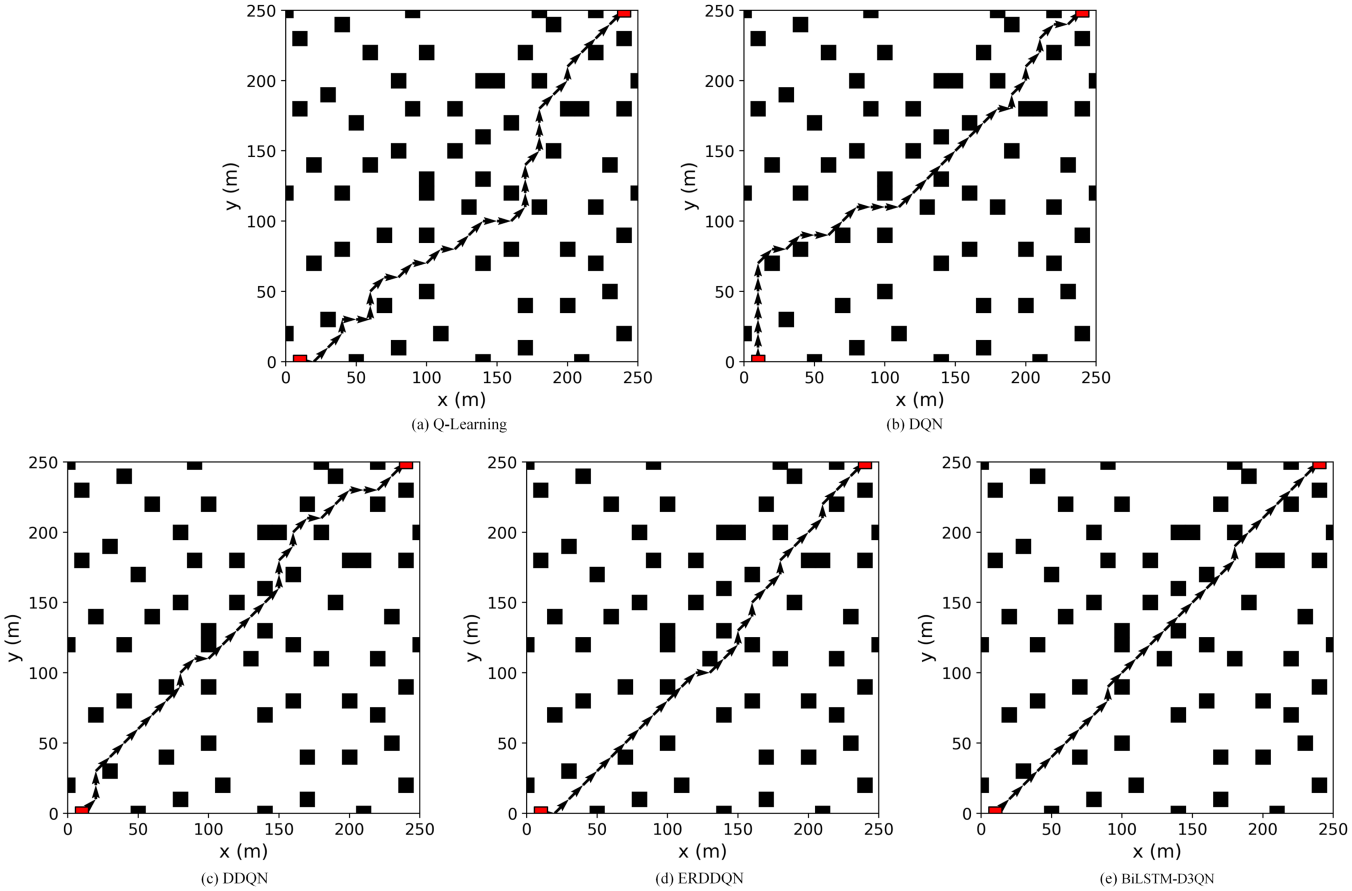
Comparison of the five algorithms for planning paths in env 2. **(a)** Q-Learning algorithm; **(b)** DQN algorithm; **(c)** DDQN algorithm; (d) ERDDQN algorithm; **(e)** BilSTM-D3QN algorithm.

**Figure 15:**
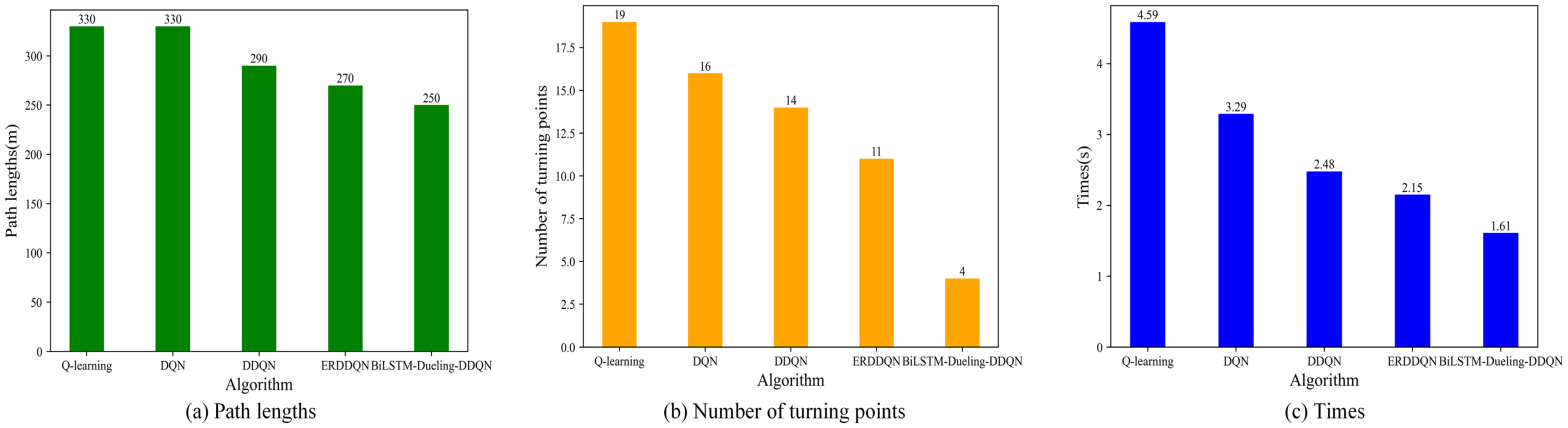
Comparison of the five algorithms indicators in env 2. **(a)** Path lengths; **(b)** Number of turning points; **(c)** Times.

Q-learning, DQN, DDQN, and ERDDQN are made to compare with the BiLSTM-D3QN algorithm proposed in this paper under environment 2. Figure 14 shows the path planning line diagrams of the five algorithms, and Figure 15 shows the comparison of the five algorithms in environment 2 in terms of various metrics, including the length of the planned path, the number of inflection points of the planned path, and the time required for path planning. From Figure 14, it can be seen that the BiLSTM-D3QN algorithm plans better paths. The worst performance of the five algorithms in the comparison is the Q-learning algorithm, the Q-learning algorithm plans a path length of 330 m, the number of turning points is 19, and the planning time is 4.59 s. The Q-learning has the most turning points, which indicates that its path planning is more unstable, there are more unnecessary path adjustments the computational complexity is higher and the optimization performance is poor. DQN and DDQN algorithms plan path lengths of 330 m and 290 m, the number of turning points is 16 and 14, and the planning time is 3.29 s and 2.48 s, respectively. The planned path length of BiLSTM-D3QN algorithm is 250 m, which is 20 m shorter than that of ERDDQN algorithm; the number of inflection points in the path of the ERDDQN algorithm is 11, which is a lot of inflection points, whereas the number of inflection points in the path of this paper’s algorithm is only 4; and this paper’s algorithm takes a shorter time for path planning, which is only 1.61 s shorter than that of the ERDDQN algorithm. Only 1.61 s is lower than the 2.15 s of ERDDQN algorithm. It shows that it is the most computationally efficient, and can converge more quickly in environmental decision making. In summary, the BiLSTM-D3QN algorithm proposed in this paper outperforms the traditional DRL algorithms and the ERDDQN algorithm in the performance of path planning.

Table 3 shows that in the more complex environment 2, the algorithm with the highest overall success rate is still the BiLSTM-D3QN algorithm proposed in this paper, which reaches 94.0%; followed by the ERDDQN algorithm at 83.6%, the DDQN algorithm at 79.2%, and the DQN algorithm at 75.2%; the overall success rate of the ordinary Q-learning algorithm is 72.8%, reflecting its poor adaptability to complex environments. Its poor ability to adapt to complex environments. BiLSTM-D3QN combines the BiLSTM network, which can not only memorize the past state but also consider the future state. It also processes the environment information more comprehensively, so it performs the best in the path planning task. In terms of growth, BiLSTM-D3QN consistently leads in the development of cumulative successes per 50 episodes, followed by ERDDQN, indicating that these two algorithms are the most capable of learning. BiLSTM-D3QN and ERDDQN have the most stable growth rates while Q-learning and DQN show more fluctuating performance. In the last episode (200–250 rounds), the growth rate of BiLSTM-D3QN increases significantly, indicating that it has a stronger ability to adapt to complex environments at a later stage. BiLSTM-D3QN not only grows the most in each episode but also grows at a relatively smooth rate, showing superior learning performance and adaptability to the environment.

**Table 3:** Cumulative number of successes in env 2.

Episode	Q-learning	DQN	DDQN	ERDDQN	BiLSTM-D3QN
50	25	32	33	37	43
100	55	69	74	79	90
150	99	107	113	120	139
200	140	146	150	163	185
250	182	188	198	209	235
Success rate	72.80%	75.20%	79.20%	83.60%	94.00%

**Figure 16:**
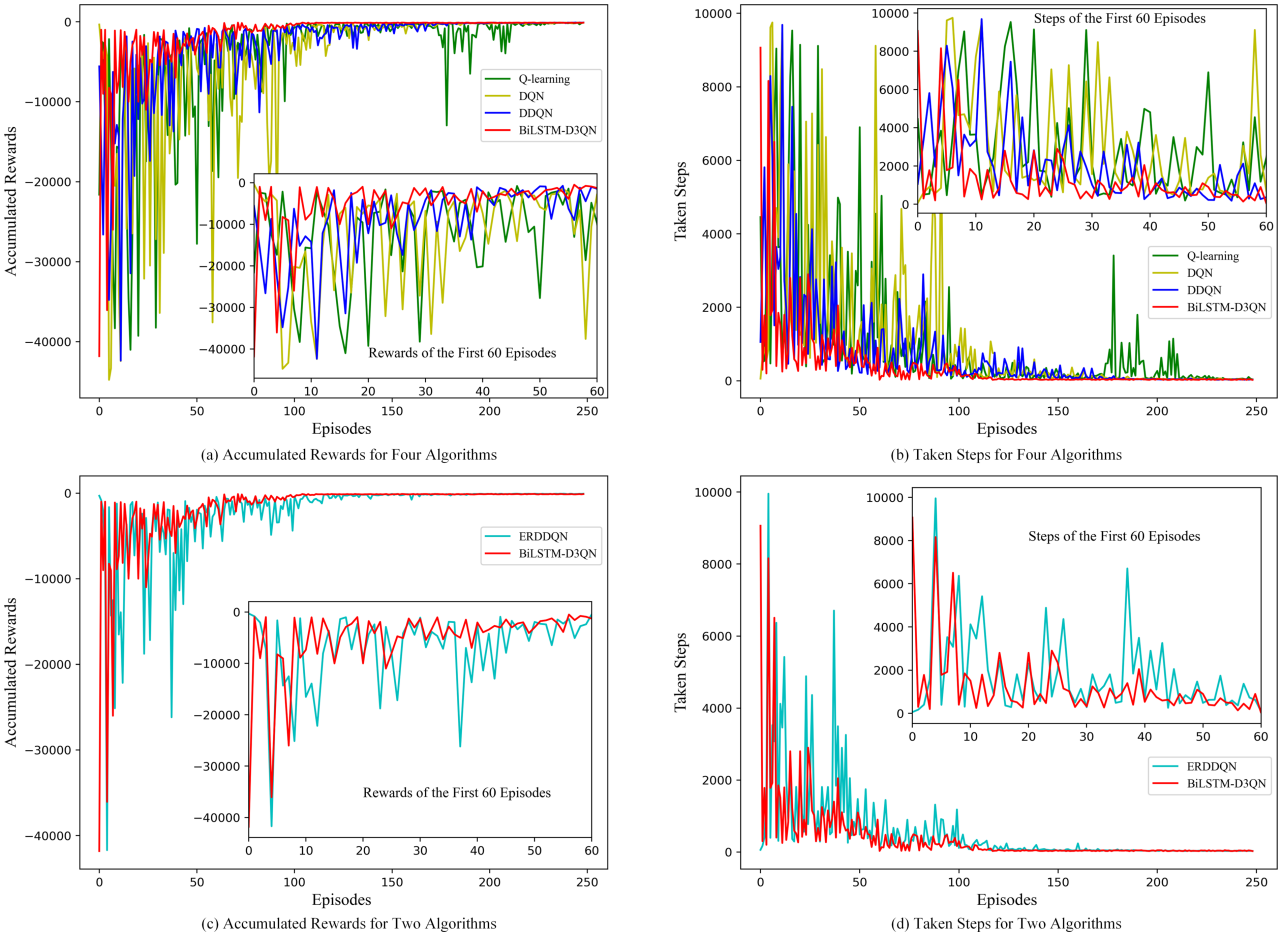
Comparison of rewards and steps accumulated per episode in env 2. **(a)** Accumulated rewards for four algorithms; **(b)** Taken steps for four algorithms; **(c)** Accumulated rewards for two algorithms; **(d)** Taken steps for two algorithms.

Figure 16 compares the accumulated rewards and steps per round of path planning performed by the robot using Q-learning, DQN, DDQN, and ERDDQN with the BiLSTM-D3QN algorithm proposed in this paper under Environment 2, where green is Q-learning, yellow is DQN, blue is DDQN, cyan is ERDDQN, and red is BiLSTM-D3QN. After 250 rounds of path planning under the same environment, all five algorithms are able to complete the robot’s path planning task of reaching the target point, but there is a big difference in the convergence speed and stability of the ERDDQN algorithm. The convergence speed and stability are very different. The Q-learning algorithm converges more slowly, and in the first 100 episodes of training, the reward value fluctuates dramatically and is still in the stage of substantial exploration. The DQN algorithm converges better than Q-learning and gradually stabilizes after about 150 episodes. DQN shows strong exploratory behavior with large fluctuations in reward values in the first 50 episodes, and the fluctuations are relatively small after convergence. However, there are still some ups and downs. The ERDDQN algorithm gradually converges after 50 episodes of active exploration in the early stage of training and reaches the maximum value of around 120 episodes. However, the reward curve and step curve show fluctuation after convergence, which indicates that in the later stage of training, although the mobile robot arrives at the goal point, the mobile robot makes unstable decisions, resulting in the fluctuation of the reward value larger. The BiLSTM-D3QN algorithm proposed in this paper converges quickly after 40 exploration episodes, and the average reward value and the number of steps are better than the ERDDQN algorithm in the process of convergence, and the degree of fluctuation of the reward curve and the number of steps curve is smaller, which is attributed to the fact that the competitive network in BiLSTM-D3QN has a separated data flow structure, which can approximate the Q function more effectively and accurately; BiLSTM-D3QN reaches its maximum value at around 100 rounds, which is faster than the comparative ERDDQN algorithm, suggesting that the reprioritised empirical replay mechanism of this paper’s algorithm, based on the frequency penalty function, improves the use of important data and thus speeds up the convergence of the neural network; BiLSTM-D3QN has very little fluctuation in the reward value function after 100 training rounds, while the ERDDQN algorithm curve shows a small fluctuation, which is due to the fact that the BiLSTM memory network in the algorithmic structure of this paper makes the front and back actions relevant, so the robot’s decision is stable and a more stable cumulative reward is achieved during path planning. In summary, the BiLSTM-D3QN algorithm outperforms the ERDDQN algorithm in terms of convergence speed, cumulative reward, and overall stability during path planning training.

### 4.3 Analysis of ROS simulation results

**Figure 17:**
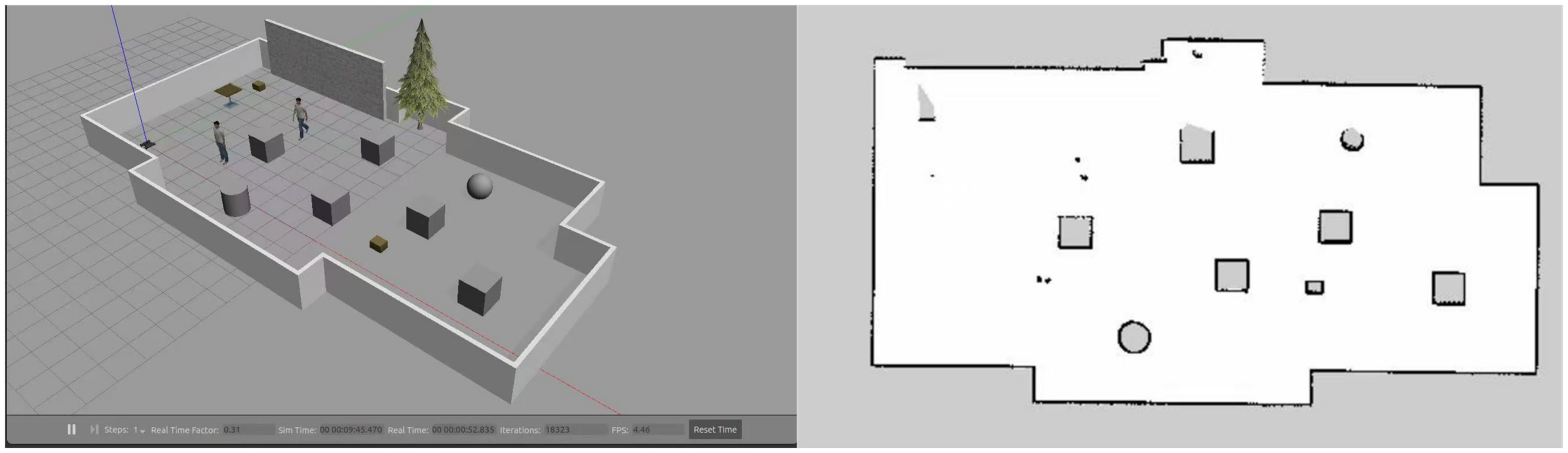
Simulation environment infographic.

**Figure 18:**
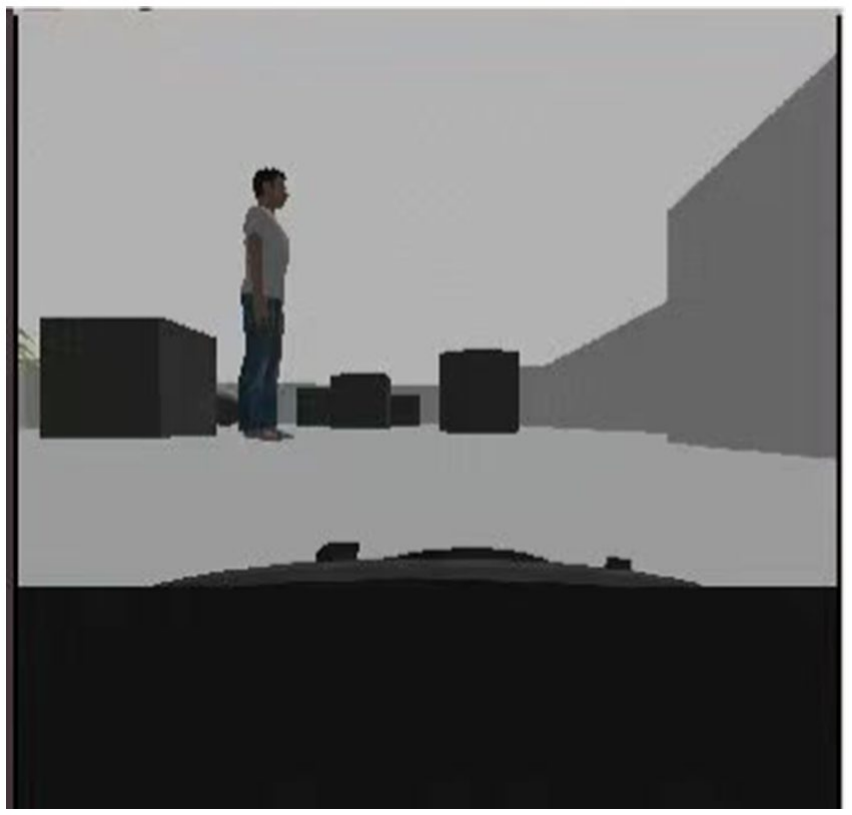
First view of a mobile robot.

**Figure 19:**
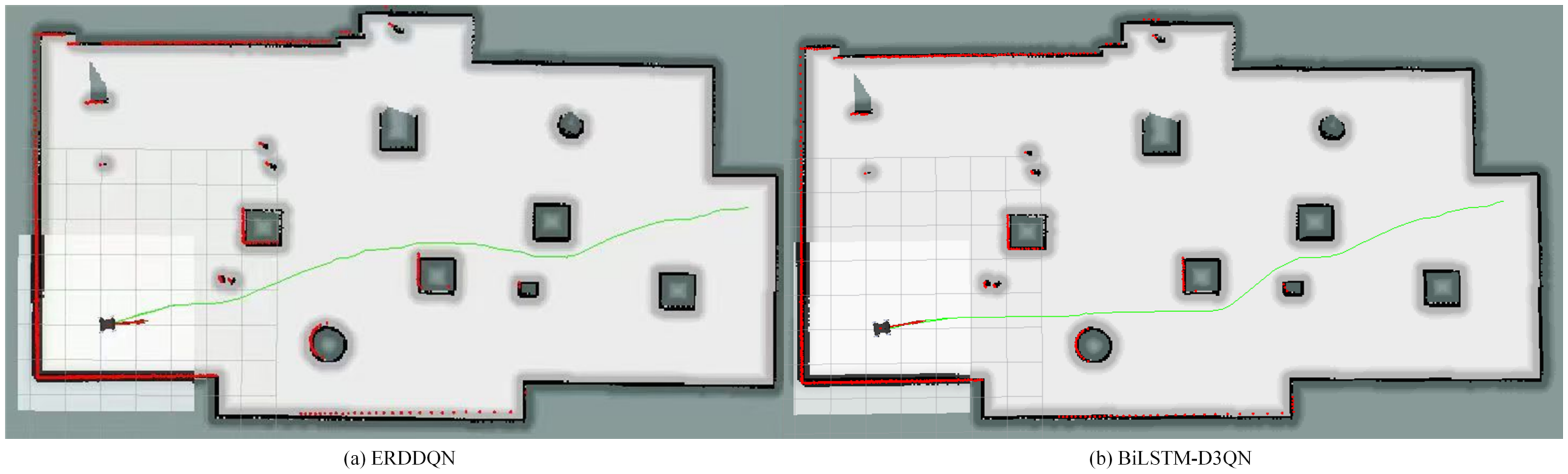
Comparison of planning paths for simulation environments. **(a)** ERDDQN algorithm; **(b)** BilSTM-D3QN algorithm.

To verify the feasibility of the improved algorithm in real robots, this section implements mobile robot path planning under the ROS Gazebo simulator. A small four-wheeled all-terrain robot of Jackal UGV is used in this experiment, and the robot autonomously builds a map of the environment by LiDAR, and the blue line is the LiDAR scanning line, as shown in Figure 17. The first view of the mobile robot during the planning process is shown in Figure 18. The ERDDQN algorithm and the BiLSTM-D3QN algorithm proposed in this paper are applied to this environment, and the actual environment path planning results obtained are shown in Figure 19. As can be seen from Figure 19, both algorithms can generate a global path in this environment. The ERDDQN algorithm is compared with the BiLSTM-D3QN algorithm proposed in this paper, and the experimental results are shown in Table 4.

**Table 4:** Path planning method results comparison.

Algorithm	Target reached	Path length (m)	Planning time (s)
ERDDQN	Yes	30.1	4.37
BiLSTM-D3QN	Yes	28.3	3.19

As shown in Table 4, the path length of the BiLSTM-D3QN algorithm proposed in this paper is 28.3 m and the path planning time is 3.19 s. Compared with the ERDDQN algorithm, the robot path length and path planning time are reduced by 5.98 and 27%, respectively, which fully verifies that in real complex environments, the BiLSTM network and the improved prioritized experience replay mechanism enable the robot to improve the collision avoidance and goal point finding ability, and reduces the robot path length and plans a better path.

## 5 Conclusion

This paper proposes the BiLSTM-D3QN path planning algorithm to improve the traditional DDQN algorithm for the path planning problem of mobile robots. On the one hand, this paper introduces a frequency penalty function, which makes the real-time important data in the experience pool fully utilized to improve the convergence rate of the neural network; on the other hand, this paper adds a competitive network architecture with separate data streams, which can approximate the Q function more effectively and accurately, and further solves the problem of overestimation of the Q-value function; The bidirectional long and short-term memory network is added to the network structure so that the model has the function of extracting and remembering the obstacle information, which improves the stability of the mobile robot’s decision making and makes the reward convergence more stable; and this paper introduces an adaptive action selection mechanism to further optimize the action exploration. Finally, simulation comparison experiments are set up in both simple and complex environments, and the experimental results show that the BiLSTM-D3QN path planning algorithm is better than the traditional deep reinforcement learning algorithm in terms of network convergence speed, planning efficiency, stability of reward convergence and success rate in simple environments; in complex environments, the path length of BiLSTM-D3QN is 20 m shorter and the number of turning points is 7% less than that of the ERDDQN algorithm. 20 m, 7 fewer turning points, 0.54 s less planning time, and a 10.4% higher success rate than the ERDDQN algorithm. The algorithm is ported to a ROS robot, and mobile robot path planning experiments are designed under the ROS Gazebo simulator, which verifies that the improved algorithm is feasible in the real world. However, since this paper only studies static obstacle avoidance and does not consider the presence of dynamic obstacles, the direction of future research is to add dynamic obstacles in the environment so that it can still obtain a better path in a more complex environment.

## Data Availability

The raw data supporting the conclusions of this article will be made available by the authors, without undue reservation.

## References

[ref1] Chen L. Wang Q. Deng C. Xie B. Tuo X. Jiang G. (2024). Improved double deep Q-network algorithm applied to multi-dimensional environment path planning of hexapod robots. Sensors 24:2061. doi: 10.3390/s24072061, PMID: 38610271 PMC11013983

[ref2] Chuanbo W. Wangneng Y. Guangze L. Weiqiang L. (2023). Deep reinforcement learning with dynamic window approach based collision avoidance path planning for maritime autonomous surface ships. Ocean Eng. 284:115208. doi: 10.1016/j.oceaneng.2023.115208, PMID: 39854647

[ref3] Debnath D. Vanegas F. Boiteau S. Gonzalez F. (2024). An integrated geometric obstacle avoidance and genetic algorithm TSP model for UAV path planning. Drones 8:302. doi: 10.3390/drones8070302

[ref4] Deguale A. D. Yu L. Sinishaw L. M. Li K. (2024). Enhancing stability and performance in Mobile robot path planning with PMR-dueling DQN algorithm. Sensors 24:1523. doi: 10.3390/s24051523, PMID: 38475059 PMC10934465

[ref5] Guo S. Zhang X. Zheng Y. du Y. (2020). An autonomous path planning model for unmanned ships based on deep reinforcement learning. Sensors 20:426. doi: 10.3390/s20020426, PMID: 31940855 PMC7013856

[ref6] Huiyan H. Jiaqi W. Liqun K. Xie H. Hongxin X. (2023). Improved robot path planning method based on deep reinforcement learning. Sensors (Basel, Switzerland) 23. doi: 10.3390/s23125622 PMC10304367 37420785

[ref7] Jinduo Z. Zhigao G. Jiakai L. Chao W. Keqiang Y. Wenjun L. (2022). Path planning research of a UAV Base station searching for disaster victims’ location information based on deep reinforcement learning. Entropy 24:1767. doi: 10.3390/e24121767, PMID: 36554172 PMC9778616

[ref8] Junli G. Weijie Y. Jing G. Zhongjuan L. (2020). Deep reinforcement learning for indoor Mobile robot path planning. Sensors (Basel, Switzerland) 20:5493. doi: 10.3390/s20195493, PMID: 32992750 PMC7582363

[ref9] Kong X. Zhou Y. Li Z. Wang S. (2024). Multi-UAV simultaneous target assignment and path planning based on deep reinforcement learning in dynamic multiple obstacles environments. Front. Neurorobot. 17:171302898. doi: 10.3389/fnbot.2023.1302898, PMID: 38318422 PMC10839049

[ref10] Lei W. Xiaodong H. Junguo C. Chao L. Wensheng X. (2023). Modified adaptive ant colony optimization algorithm and its application for solving path planning of mobile robot. Expert Syst. Appl. 215:119410. doi: 10.1016/j.eswa.2022.119410, PMID: 39854647

[ref11] Li Q. Geng X. (2023). Robot path planning based on improved DQN algorithm. Comput. Eng. 49, 111–120. doi: 10.19678/j.issn.1000-3428.0066348

[ref12] Lin Y. Wen J. (2023). Improved duelling deep Q-networks based path planning for intelligent agents. Int. J. Veh. Des. 91, 232–247. doi: 10.1504/IJVD.2023.131056, PMID: 35009967

[ref13] Meetu J. Vibha S. Narinder S. Satya Bir S. (2022). An overview of variants and advancements of PSO algorithm. Appl. Sci. 12:8392. doi: 10.3390/app12178392, PMID: 39838284

[ref14] Meng X. Jiachen Y. Jiabao W. Zhengjian L. Wen L. Xinbo G. (2023). An information-assisted deep reinforcement learning path planning scheme for dynamic and unknown underwater environment. IEEE Trans. Neural Netw. Learn. Syst. 36, 842–853. doi: 10.1109/TNNLS.2023.3332172 37988205

[ref15] Sha L. Mingyue Z. Yongbo Z. Cheng M. Qingdang L. (2023). A survey of path planning of industrial robots based on rapidly exploring random trees. Front. Neurorobot. 17:171268447. doi: 10.3389/fnbot.2023.1268447 PMC10654791 38023457

[ref16] Shen X. Zhao T. (2023). UAV regional coverage path planning strategy based on DDQN. Electron. Meas. Technol. 46, 30–36. doi: 10.19651/j.cnki.emt.2211675

[ref17] Tang J. Liang Y. Li K. (2024). Dynamic scene path planning of UAVs based on deep reinforcement learning. Drones 8:60. doi: 10.3390/drones8020060

[ref18] Wang X. Zhong W. Wang J. Xiao L. Zhu Q. (2024). UAV path and radio mapping based on deep reinforcement learning. J. Appl. Sci. 42, 200–210. doi: 10.3969/j.issn.0255-8297.2024.02.002

[ref19] Yan Y. Zhiyu C. Gang L. Jianwei G. (2023). A Mapless local path planning approach using deep reinforcement learning framework. Sensors 23:2036. doi: 10.3390/s23042036, PMID: 36850635 PMC9958619

[ref20] Yu X. Yang L. Yubo T.. (2024). A* algorithm based on adaptive expansion convolution for unmanned aerial vehicle path planning. Intell. Serv. Robot. 17:521. doi: 10.1007/s11370-024-00536-3

[ref21] Yuan S. Zhang L. Gu Q. Zhang F. Lv J. (2023). Research on D3QN path planning method of Mobile robot priority sampling. J. Chin. Comput. Syst. 44, 923–929. doi: 10.20009/j.cnki.21-1106/TP.2021-0713

[ref22] Yuwan G. Zhitao Z. Jidong L. Lin S. Zhenjie H. Shoukun X. (2022). DM-DQN: dueling Munchausen deep Q network for robot path planning. Complex Intell. Syst. 9, 4287–4300. doi: 10.1007/S40747-022-00948-7

[ref23] Zhang Y. Liu K. Gao F. Zhao F. (2023). Research on path planning and path tracking control of autonomous vehicles based on improved APF and SMC. Sensors 23:7918. doi: 10.3390/s23187918, PMID: 37765974 PMC10535914

[ref24] Zhao W. Zhang Y. Xie Z. (2024). EPPE: an efficient progressive policy enhancement framework of deep reinforcement learning in path planning. Neurocomputing 596:127958. doi: 10.1016/j.neucom.2024.127958, PMID: 39854647

[ref25] Zhou X. Yan J. Yan M. Mao K. Yang R. Liu W. (2023). Path planning of rail-mounted logistics robots based on the improved Dijkstra algorithm. Appl. Sci. 13:9955. doi: 10.3390/app13179955

